# Design, preparation and application of the semicarbazide-pyridoyl-sulfonic acid-based nanocatalyst for the synthesis of pyranopyrazoles

**DOI:** 10.1038/s41598-022-18651-5

**Published:** 2022-08-23

**Authors:** Masoumeh Beiranvand, Davood Habibi

**Affiliations:** Department of Organic Chemistry, Faculty of Chemistry, Bu-Ali Sina University, Hamedan, Iran

**Keywords:** Chemistry, Nanoscience and technology

## Abstract

A novel, efficient, and recoverable nanomagnetic catalyst bearing the semicarbazide linkers, namely, Fe_3_O_4_@SiO_2_@OSi(CH_2_)_3_-*N*(3-pyridoyl sulfonic acid)semicarbazide (FSiPSS) was designed, synthesized and characterized by the use of various techniques such as FT‐IR, EDX, elemental mapping analysis, XRD, SEM, TEM, TGA/DTA, BET, and VSM. Then, the catalytic capability of the novel prepared nanomagnetic FSiPSS catalyst was successfully investigated in the synthesis of diverse pyranopyrazoles through a one-pot four-component condensation reaction of ethyl acetoacetate, hydrazine hydrate, aromatic aldehydes, and malononitrile or ethyl cyano-acetate by the help of ultrasonication in very short reaction time, good to high yields and easy work-up (Fig. 1).

**Figure 1:**
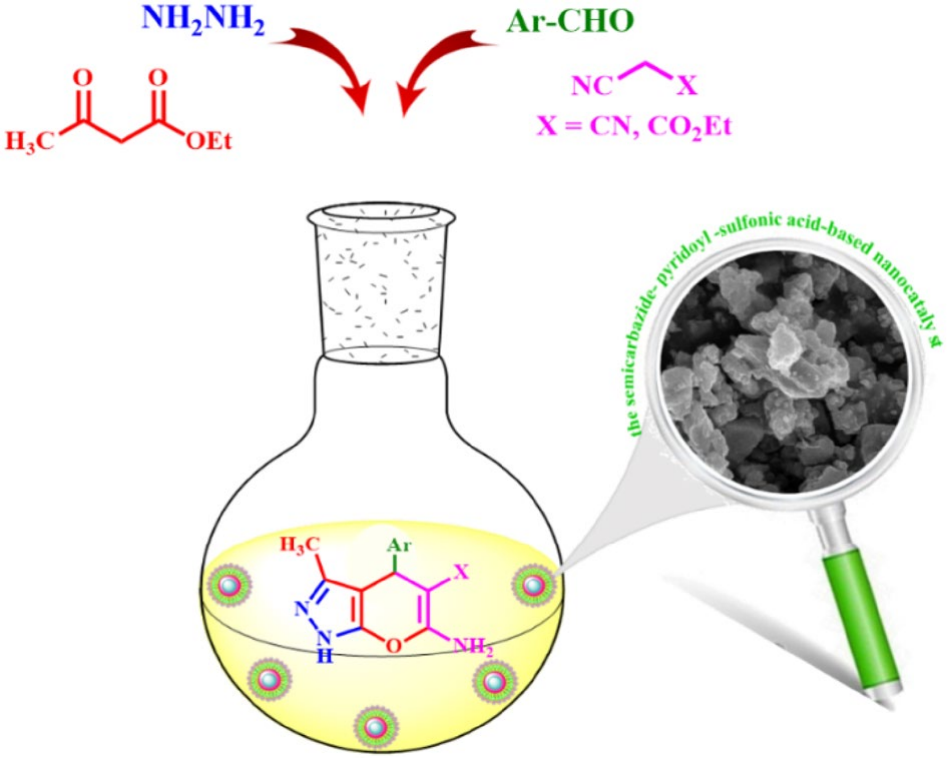
Synthesis of diverse pyranopyrazoles by the FSiPSS nano-catalyst.

Synthesis of diverse pyranopyrazoles by the FSiPSS nano-catalyst.

## Introduction

Semicarbazide (SEM) is a derivative of urea or hydrazine that possess several important functions in medicinal and health-related issues. SEM motifs constitute the core structures of several drugs and herbicides such as nitrofurazone, tolazamide, laromustine, cafenstrole, and diflufenzopyr^1–3^. In addition, SEM is applied in food as a marker to detect the illegal usage of the banned antibiotic nitrofurazone^4^. They also reveal a stabilizing effect on the liquid crystalline state of chloroplast membrane lipids, and some are known as surfactants. Another report exhibited that SEMs are also applied as stabilizing agents in the polymer industry^5^.

Also, magnetic nanoparticles (MNPs) are receiving increasing interest due to their widespread applications in various fields. MNPs have many advantages in organic chemistry, (1) MNPs are accessible; (2) the stability of catalyst linkages leads to the use of more environmentally friendly solvents than homogeneous catalysis; (3) simple separation by an external magnetic field; (4) the fabrication of MNPs is generally simple, scalable, safe, cost-effective and controllable; (5) catalyst leaching is usually lower than other material-supported catalysts^6^. Many reports on MNPs nanoparticles have appeared over during years^7–11^. Among different types of MNPs, aluminum and iron oxide have large advantages such as low cost, extensive availability, thermal stability, and considerable adsorption capacity^12^.

In particular, iron oxide nanoparticles (IONPs), which belong to the ferrimagnetic class of magnetic materials, are wildly applied in the fields of biomedicine and bioengineering due to their ease of surface modification, synthesis, and low toxicity. Magnetite (Fe_3_O_4_) and maghemite (γ-Fe_2_O_3_) and mixed ferrites (MFe_2_O_4_ where M=Co, Mn, Ni, or Zn) are the three main forms of iron oxide-based nanoparticles^13–16^. To prevent the aggregation of MNPs and also enhancement their stability of them, usually, a layer of silica is coated on the surface^17^. Fe_3_O_4_ coated with silica was often used as the support of metal and nonmetal catalysts^18–21^.

Magnetic nanomaterials are more efficient adsorbents than active carbon, graphene oxide (GO), and zeolite-based adsorbents due to their ease of removal of contaminants from wastewater employing an applied magnetic field but also their advantageous surface charge and redox activity characteristics. The incorporation of magnetic nanomaterials with adsorbents such as WO_3_, TiO_2_, ZnO, and GO decreases the rapid recombination of photoinduced electron holes and improves the photocatalysis potential of these materials. On the other hand, magnetic nanomaterials can a synergistic effect with biosorbents. Biosorbents possess efficient adsorption capacity to eliminate polluters, and high abundance and therefore help diminish ecological and environmental problems^22,23^.

Among iron oxide magnetic nano-particles, sulfonic acid-functionalized magnetic nanoparticles, known as the recoverable solid strong acid, have attracted much attention due to economically important and environmentally benign features^24^.

In addition, pyranopyrazoles (six-membered oxygen-containing heterocycles) have received considerable attention due to the wide range of biological activities such as anti-cancer, anti-leishmanial, antimicrobial, anti-inflammatory, lactamase inhibitor, etc.^25–28^. Three-component (3-CR) or four-component (4-CR) reactions are often used for the synthesis of pyranopyrazoles^29^. Several methods have been established for their synthesis using copper-immobilized ionic liquid^30^, *N*-methylmorpholine *N*-oxide and silver oxide (Ag_2_O)^31^, isonicotinic acid^32^, cetyltrimethylammonium chloride (CTACl)^33^, [bmim]BF_4_^34^, choline chloride-urea deep eutectic solvent^35^, bael fruit ash (BFA)-catalyst^36^, P_2_O_5_/SiO_2_ or H_3_PO_4_/Al_2_O_3_^37^, Nd-salen Schiff base complex immobilized mesoporous silica^38^, uncapped SnO_2_ quantum dots (QDs)^39^, sodium citrate^40^, trityl carbocation^41^, CeO_2_/ZrO_2_^42^, saccharose^43^, per-6-amino-β-cyclo-dextrin (per-6-ABCD)^44^, 2-carboxy-*N*,*N*-diethylethan-aminium acetate^45^, cinchona alkaloid^46^, 4CzIPN/Ni^0^-metallaphotoredox^47^, sodium ascorbate^48^ and Meglumine^49^.

**Figure 2:**
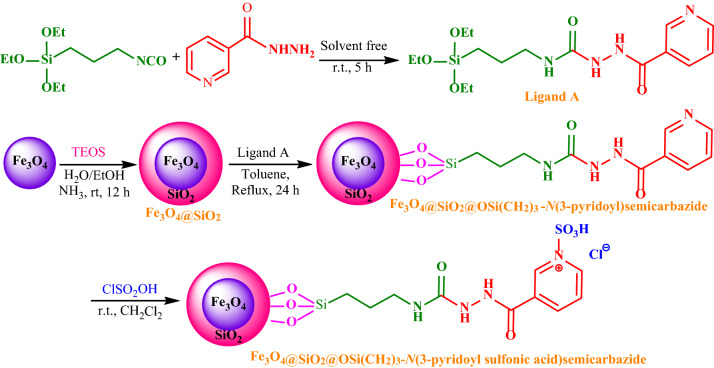
Synthesis of the FSiPSS nano-catalyst.

In this paper and following our interests to present new and efficient protocols for the synthesis of biological valuable structures by the use of nanomagnetic catalysts^50–54^, we would like to report the rational design, synthesis, and characterization of the novel FSiPSS nano-catalyst (Fig. 2).

**Figure 3:**
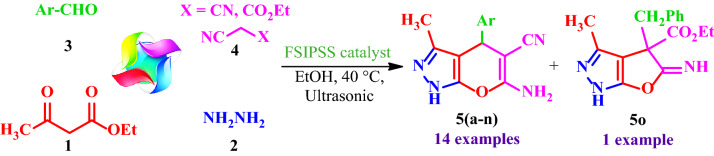
Synthesis of pyranopyrazoles.

Then, the FSiPSS nano-catalyst was used as an efficient heterogeneous catalyst for the synthesis of pyranopyrazoles via a one-pot four-component condensation reaction of ethyl acetoacetate **1**, hydrazine hydrate **2**, aromatic aldehydes **3**, and malononitrile or ethyl-cyano-acetate **4** under ultrasonic conditions (Fig. 3).

## Experimental

### General

All the commercial reagents were obtained from the Merck or Aldrich chemical companies and used without further purification. The reaction progress and purity of the synthesized compounds were monitored by TLC performed with silica gel 60 F-254 plates. FT‐IR spectra were recorded on a PerkinElmer Spectrum Version 10.02.00 using KBr pellets. The ^1^H NMR (250 MHz) and ^13^C NMR (62.5 MHz) spectra were recorded on a Bruker spectrometer (δ in ppm) using DMSO‐d_6_ as a solvent with chemical shifts measured relative to TMS as the internal standard. Melting points were taken with a BUCHI 510 melting point apparatus. Elemental analysis was done using a MIRA II analyzer. The TEM images were recorded on a CM120 total carbon analyzer. The FESEM images were recorded using a MIRA III analyzer. The X-ray diffraction (XRD) measurements were performed with an XRD Philips PW1730. Thermogravimetric-differential thermal analysis (TG–DTA) was carried out using an SDT-Q600 device. A 2200 ETH-SONICA ultrasound cleaner (50 Hz) was employed for ultrasonication purposes.

A novel, efficient, and recoverable nanomagnetic catalyst bearing semicarbazide linker, namely, Fe_3_O_4_@SiO_2_@OSi(CH_2_)_3_-*N*(3-pyridoyl sulfonic acid)semicarbazide was designed, synthesized, and characterized using various techniques including Fourier transform infrared (FT‐IR) spectroscopy, energy dispersive X‐ray (EDX) analysis, elemental mapping analysis, X‐ray diffraction (XRD), scanning electron microscopy (SEM), transmission electron microscopy (TEM), thermo-gravimetric analysis/differential thermal analysis (TGA/DTA), vibrating sample magnetometer (VSM) and Brunauer–Emmett–Teller (BET).

### General procedure for preparation of the ligand A

Initially, ligand A (2-nicotinoyl-*N*-(3-(triethoxysilyl)propyl)hydrazine-1-carboxamide) was prepared through the reaction of triethoxy(3‐isocyanatopropyl)silane (1.237 g, 5 mmol) and nicotinic acid hydrazide (685 mg, 5 mmol) under solvent-free conditions at room temperature for 5 h. Then, the resulting pale-yellow precipitate was washed with a mixture of *n*‐hexane and dichloromethane (3 × 10 mL). The obtained product was air-dried and characterized using FT‐IR, ^1^H NMR, and ^13^C NMR.

### General procedure for the construction of Fe_3_O_4_@SiO_2_@OSi(CH_2_)_3_-*N*(3-pyridoyl sulfonic acid)semicarbazide (FSiPSS)

The FSiPSS nano-catalyst was prepared in the following four consecutive stages:

**Stage 1:** The Fe_3_O_4_ magnetic nanoparticles (MNPs) were prepared based on the literature^55^. The mixture of FeCl_3_∙6H_2_O (11.44 g, 42.39 mmol) and FeCl_2_·4H_2_O (4.3 g, 21.62 mmol) was dissolved in water (100 mL) and stirred for 30 min at 80 °C. Then, the 37% ammonia solution (20 mL) was added dropwise to the resulting mixture and heated at 70 °C with vigorous stirring in pH 10 for 0.5 h. After separation by an external super magnet, a black precipitate (Fe_3_O_4_ = F) was filtered, washed with water, and air-dried.

**Stage 2:** The surface of the obtained Fe_3_O_4_ MNPs was coated with SiO_2_ layers^56^. Fe_3_O_4_‐MNPs (2.0 g) were dispersed in a mixture of EtOH and deionized water (250 ml, V/V = 4:1) under ultrasonic conditions for 15 min. Then, NH_3_.H_2_O (3 mL) and TEOS (2 mL) were slowly added dropwise and the mixture was stirred for a further 12 h. Fe_3_O_4_@SiO_2_ (FSi) were collected by magnetic separation, washed with water and ethanol, and vacuum dried.

**Stage 3:** The surface of Fe_3_O_4_@SiO_2_ was functionalized with the ligand A. So, Fe_3_O_4_@SiO_2_ (1.0 g) was mixed with ligand A (0.768 g, 2 mmol) under refluxing anhydrous toluene for 48 h. Then, the obtained Fe_3_O_4_@SiO_2_@OSi(CH_2_)_3_-*N*(3-pyridoyl)semicarbazide (FSiPS) was separated with a super magnet, washed with ethanol, and vacuum dried.

**Stage 4:** Further functionalization of Fe_3_O_4_@SiO_2_@OSi(CH_2_)_3_-*N*(3-pyridoyl)semi-carbazide was done with chlorosulfuric (chlorosulfonic) acid (0.133 mL, 233 mg, 2 mmol) in CH_2_Cl_2_ at ice bath. Then, the precipitate (FSiPSS) was separated with a super magnet, washed with CH_2_Cl_2_ and air-dried. The structure and morphology of FSiPSS were fully confirmed by various techniques.

### General procedure for the synthesis of pyranopyrazoles catalyzed by FSiPSS

A mixture of ethyl acetoacetate **1** (0.130 g, 1.0 mmol), hydrazine hydrate **2** (0.032 g, 1.0 mmol), aromatic aldehydes **3** (1.0 mmol), malononitrile **4a** or ethyl cyanoacetate **4b** (1.5 mmol) and FSiPSS (20 mg) was heated at 40 °C under ultrasonic irradiation in EtOH (5 mL). Reaction progress was monitored by TLC (*n*-hexane/EtOAc). After completion of the reaction, the FSiPSS nano-catalyst was separated by a super magnet, the pure products obtained by recrystallization in ethanol and characterized by FT‐IR, NMR, and mass spectrometry techniques.

## Results and discussion

The formation of the FSiPSS nano-catalyst was confirmed by various techniques involving FT‐IR, EDX, VSM, XRD, SEM, TEM, TGA/DTA, and BET.

### Characterization of the FSiPSS nanocatalyst by FT‐IR

**Figure 4:**
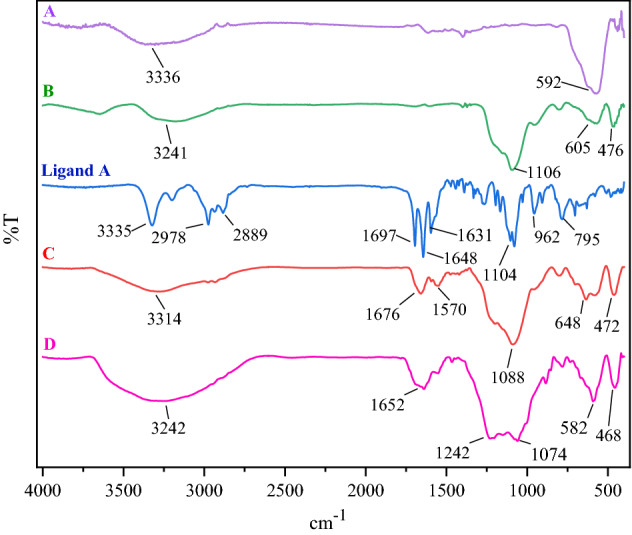
FT-IR spectra of FSiPSS and the corresponding compounds.

In a comparative exploration as indicated in Fig. 4, the FT‐IR spectra of **A**: Fe_3_O_4_, **B**: Fe_3_O_4_@SiO_2_, the ligand A, **C**: Fe_3_O_4_@SiO_2_@OSi(CH_2_)_3_-*N*(3-pyridoyl)semicarb-azide and **D**: Fe_3_O_4_@SiO_2_@OSi(CH_2_)_3_-*N*(3-pyridoyl sulfonic acid)semicarbazide were explored. The peak at about 592 cm^−1^ is related to the presence of the Fe–O stretching vibrations in the curve of Fe_3_O_4_. The FT‐IR spectrum of Fe_3_O_4_@SiO_2_ involves a new peak at 1106 cm^−1^ which is related to the Si–O–Si absorption band. FT‐IR spectrum of synthesized ligand A shows three basic characteristic peaks at 3335, 1697, and 1648 cm^−1^ indicating the presence of the NH, C=O, and C=N bonds respectively. The spectrum of Fe_3_O_4_@SiO_2_@OSi(CH_2_)_3_-*N*(3-pyridoyl)semicarbazide exhibits all above mentioned characteristic peaks. Finally, the broad peak from 2700 to 3700 cm^−1^ shows the existence of acidic OH and NH functional groups within the structure of the desired catalyst. Consequently, the comparison of all the IR spectra confirms the successful construction of the semicarbazide-pyridoyl-sulfonic acid-based nano-catalyst.

### Characterization of the FSiPSS nanocatalyst by EDX and elemental mapping analysis

**Figure 5:**
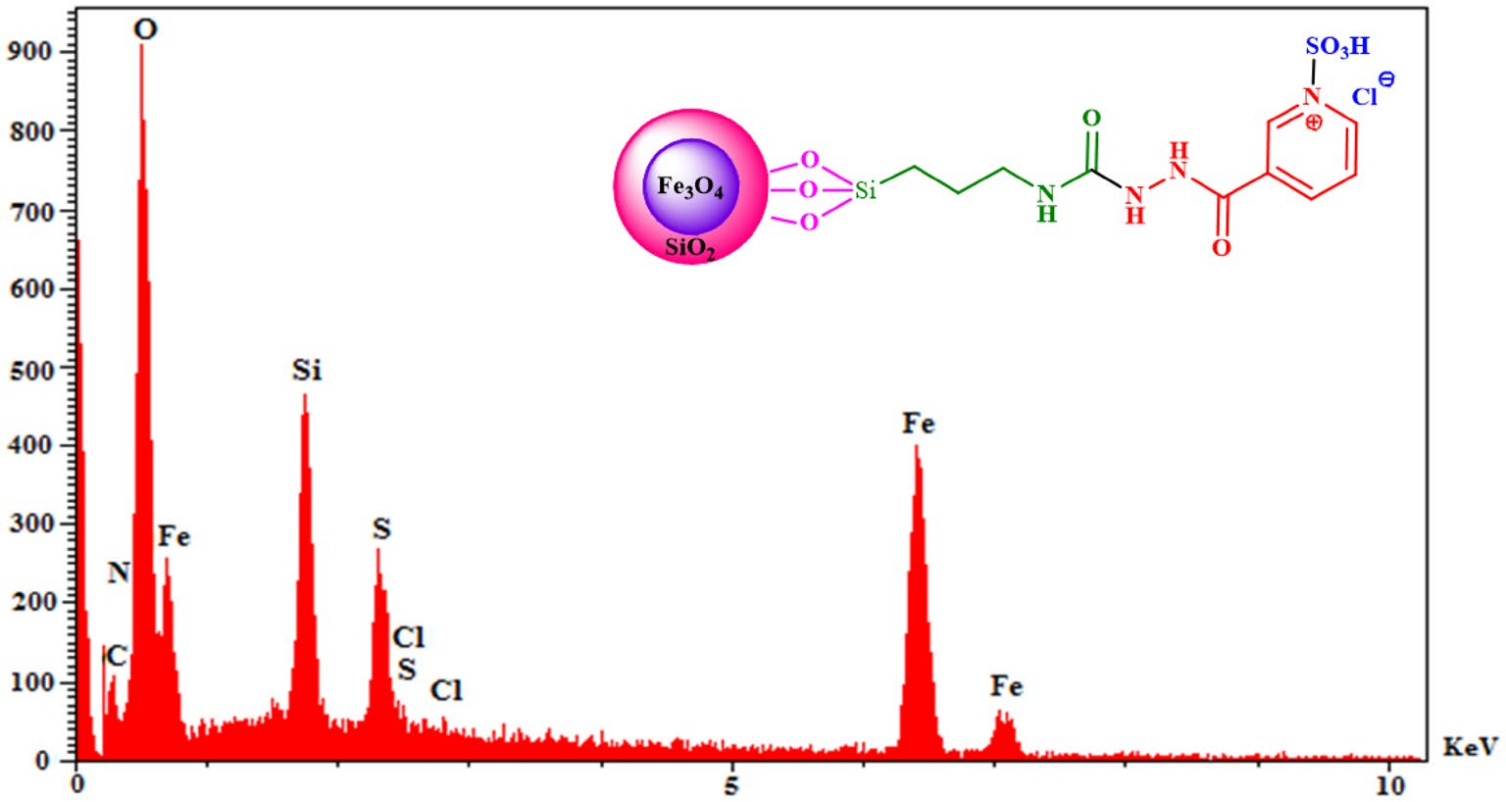
The EDX analysis of the FSiPSS nano-catalyst.

**Figure 6:**
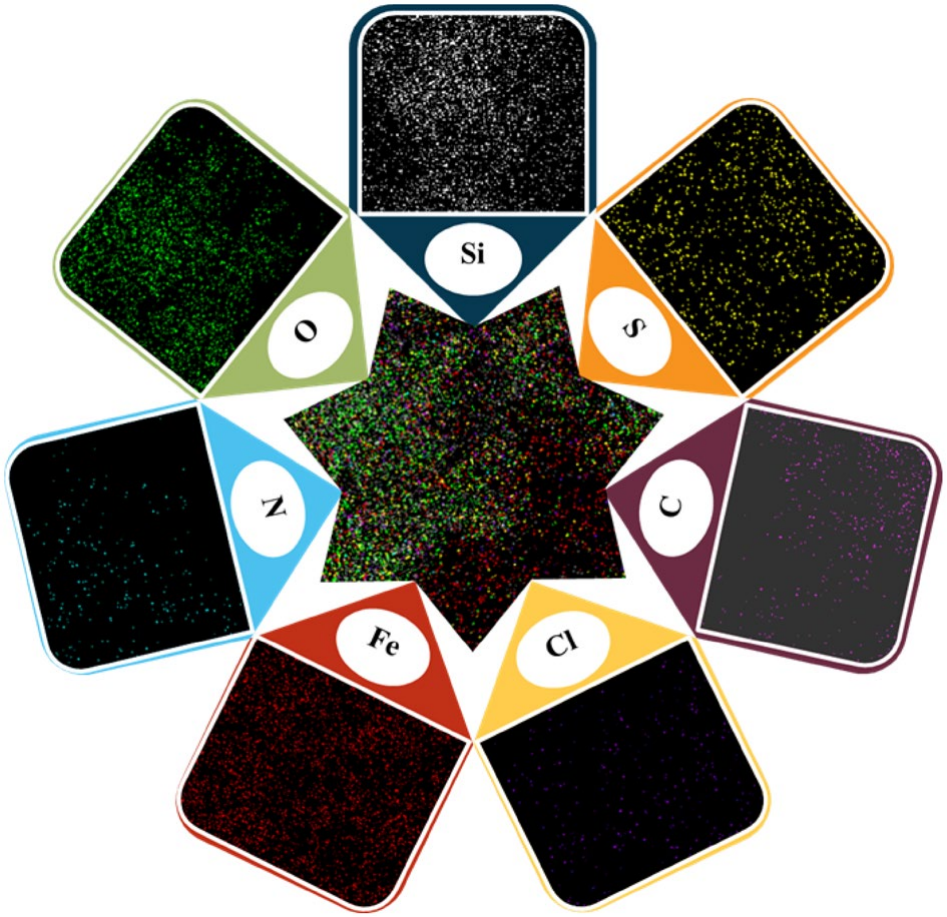
Elemental mapping analysis of the FSiPSS nano-catalyst.

As shown in Figs. 5 and 6, the obtained results from both EDX and elemental mapping analysis confirmed the existence of Fe, Si, O, C, N, S, and Cl elements in the structure of the synthesized nanocatalyst. The percentages of each element are presented in Table 1.

**Table 1:** The percentages of each element in EDX analysis.

Element	Fe	Si	O	C	N	S	Cl
W%	29.98	5.72	39.99	11.69	9.87	3.44	0.31

### Characterization of the FSiPSS nanocatalyst by VSM

**Figure 7:**
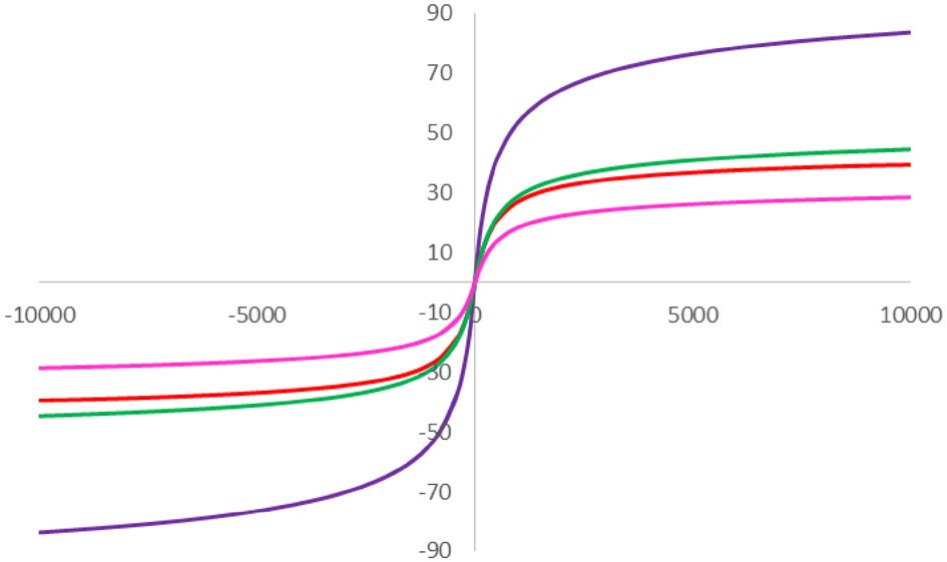
The VSM analyses of **A**, **B**, **C** and **D**.

In another study, VSM analysis was performed for the exploration of the magnetic behavior of the FSiPSS nano-catalyst (**D**) and the corresponding compounds (**A, B, C**). As illustrated in Fig. 7, decrease saturation magnetization from about 70 emu g^−1^ (for major core Fe_3_O_4_) to about 10 emu g^−1^ for the FSiPSS nano-catalyst is related to the newly coated layer which can be explained by the reduction in the dipole–dipole interactions between the magnetic nanoparticles after their coating with SiO_2_ and functionalization with ligand A and chlorosulfonic acid.

### Characterization of the FSiPSS nano-catalyst by the SEM

**Figure 8:**
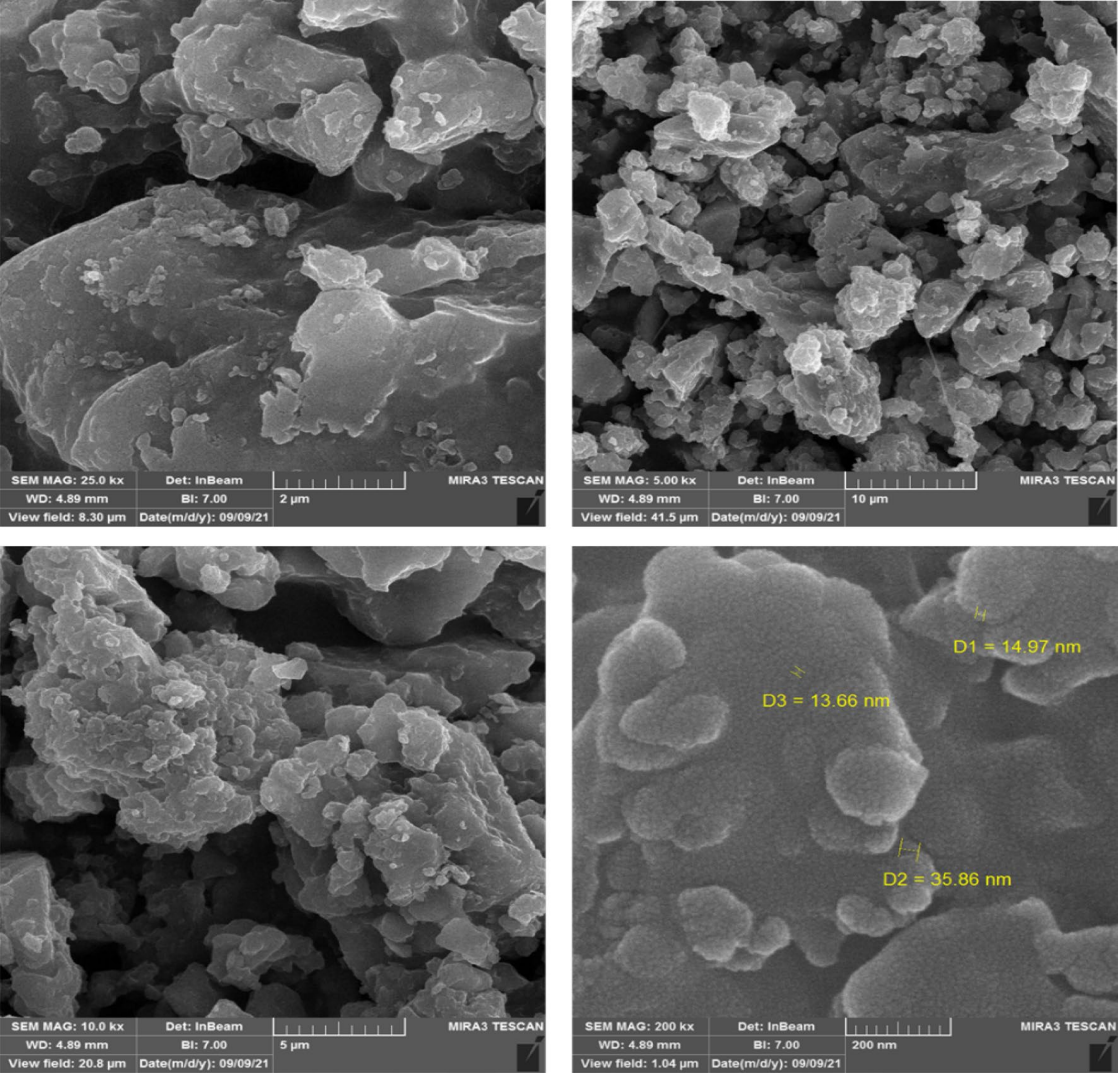
The SEM images of the FSiPSS nano-catalyst.

To study the particle size and surface morphology of the newly prepared catalyst, SEM images were also taken. The resulting images are exposed in Fig. 8. According to these images, the sizes of the FSiPSS nano‐catalyst particles are in the nanometer ranges (between 13.66 and 35.86 nm).

### Characterization of the FSiPSS nano-catalyst by the TEM images

**Figure 9:**
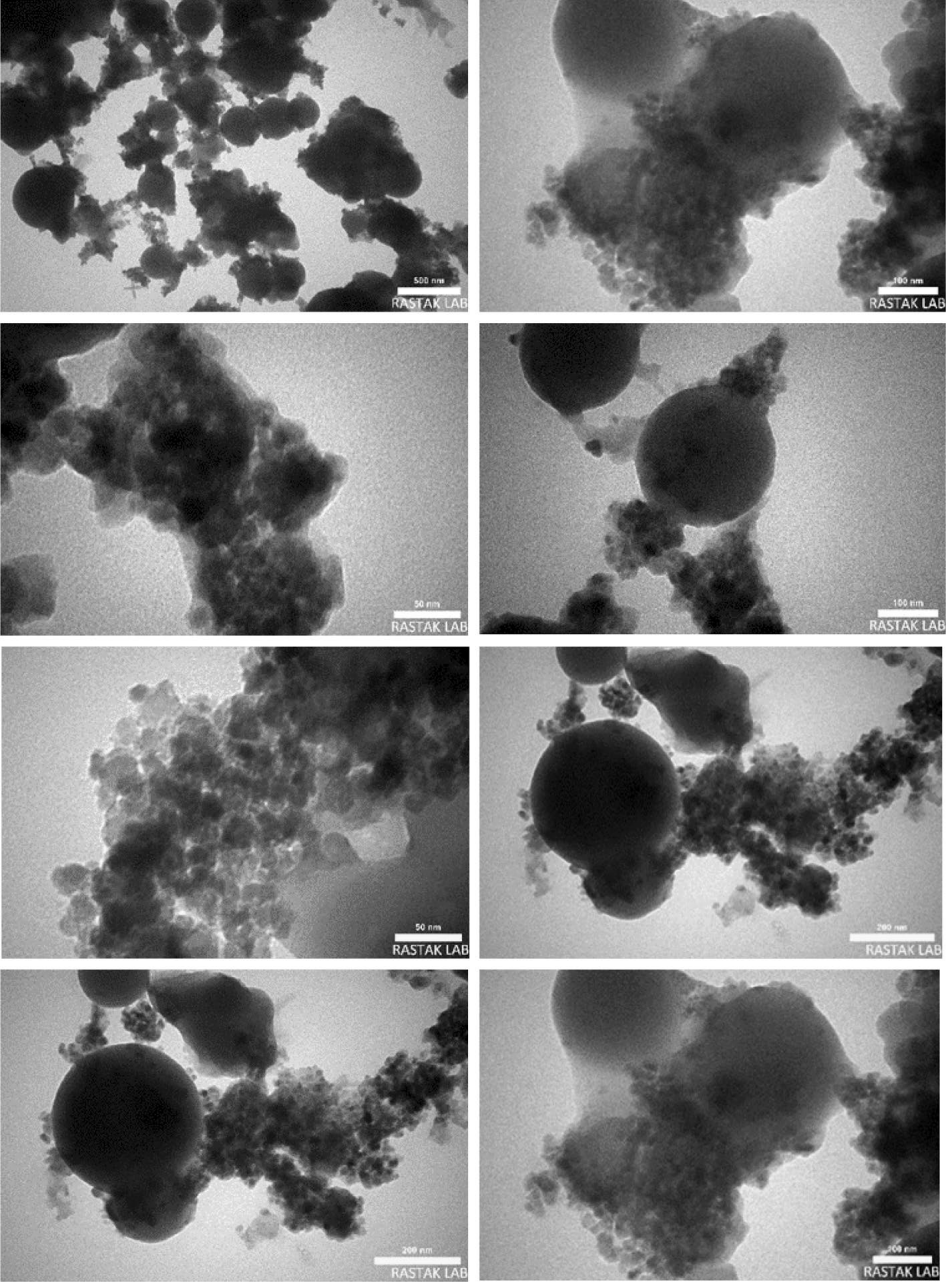
The TEM images of the FSiPSS nano-catalyst.

The obtained TEM images also proved that the sizes of the FSiPSS nano‐catalyst particles are in the nanometer ranges, as shown in Fig. 9. Moreover, the core–shell structure of the nano-catalyst can be apperceived through TEM images.

**Figure 10:**
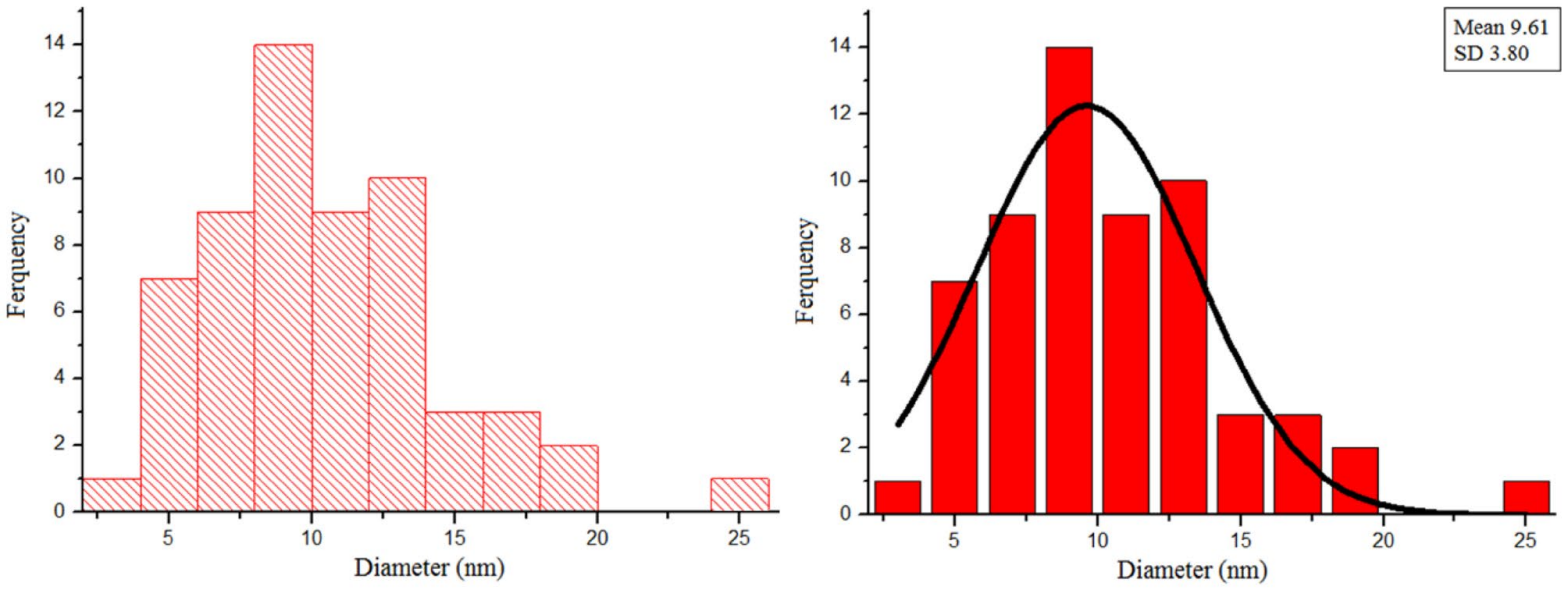
The particle size distribution (histogram) of the FSiPSS nano-catalyst.

At a closer investigation, as illustrated in the particle size distribution histograms (Fig. 10), the sizes of the nanoparticles are between 5 and 20 nm, and the average particle size is evaluated at about 9.61 nm.

### Characterization of the FSiPSS nano-catalyst by TGA-DTA

**Figure 11:**
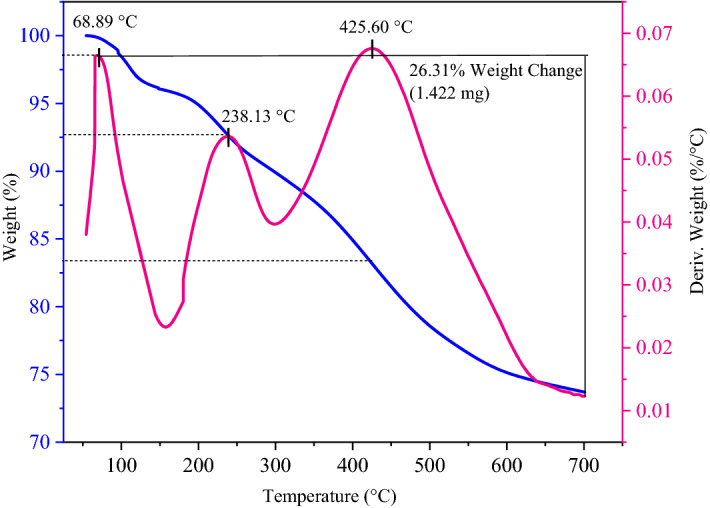
The TGA-DTA patterns of the FSiPSS nano-catalyst.

In addition, TGA-DTA analysis was applied to investigate the thermal behavior of the FSiPSS nano-catalyst. The obtained curve is presented in Fig. 11. The thermo-gravimetric curve displays the three mass losses upon heating. The weight loss from about 60–120 °C (23%) can be attributed to the loss of water molecules, the weight loss from 120 to 300 °C (7%) can be related to the decomposition of acidic functional groups and the weight loss from 300 to 650 °C (17%) can be attributed to the decomposition of the ligand A. Also, about 72% of the initial mass remains at 700 °C.

### Characterization of the FSiPSS nano-catalyst by XRD

**Figure 12:**
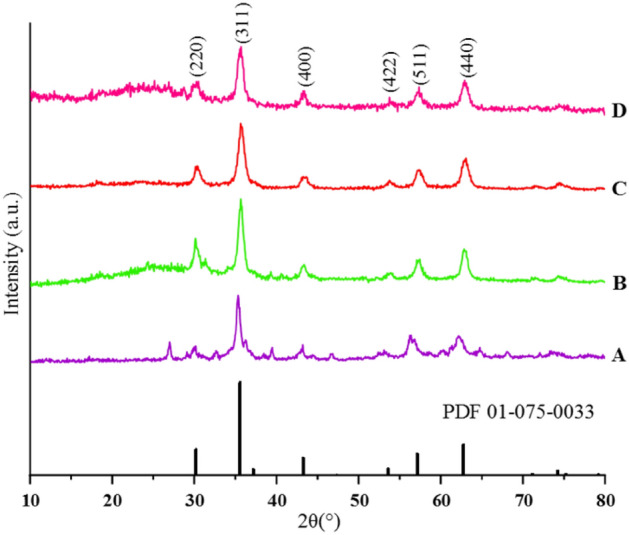
The XRD patterns of **A**, **B**, **C**, **D** and simulated pattern of the structure.

Crystalline phases and the diffraction planes of FSiPSS nanocomposites were ascertained by the XRD study, as presented in Fig. 12. FSiPSS pattern represents a single-phase profile indicating a united entity of the assembled counterparts. The typical diffraction peaks, due to the FSiPSS nano-catalyst are observed at 2θ = 30.1, 35.5, 43.1, 53.5, 57, and 62.8, corresponding to (220), (311), (400), (422), (511) and (440) Miller indices, respectively (ICDD, PDF, file no. 01-075-0033)^56^. The obtained pattern is in good agreement with the characteristic peaks of bare Fe_3_O_4_ which indicates the retention of the crystalline spinel ferrite core structure during the functionalization of MNPs and the successful synthesis of desired catalyst^57^. In addition, the successful synthesis of Fe_3_O_4_@SiO_2_ core–shell was confirmed by the presence of a broad peak at 2θ = 20°–30° which is due to the amorphous silicon layer, demonstrating that the magnetic moiety structure was protected in the core where SiO_2_ cover did not alter the crystal structure of the magnetic Fe_3_O_4_ nanoparticles^58^. In addition, after anchoring the OSi(CH_2_)_3_-*N*(3-pyridoyl sulfonic acid)semicarbazide functional groups, the peaks were found to have background noise levels increased, that coming from the amorphous added sulfonic acid functionalities^59^. Finally, based on the Scherrer equation (D = Kλ/(β cos θ), the average crystallite size of FSiPSS nanocomposites was found to be about 16.27 nm.

### Characterization of the FSiPSS nano-catalyst by BET

**Figure 13:**
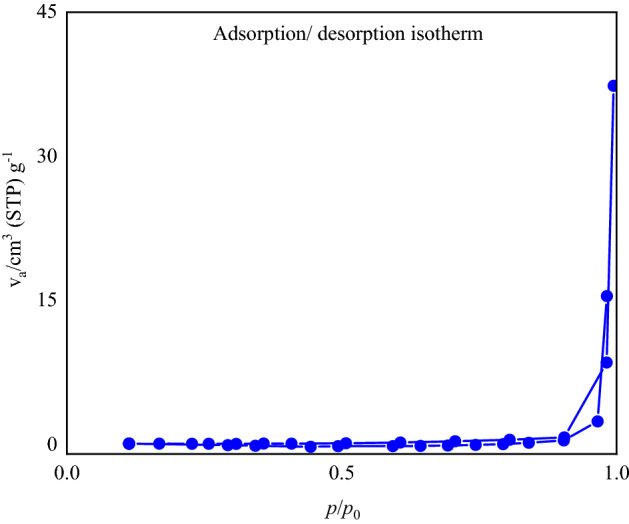
The nitrogen adsorption–desorption curve (BET) of the FSiPSS nano-catalyst.

The specific surface area of the synthesized catalyst was determined by the N_2_ adsorption–desorption analysis. The specific surface area, the total pore volumes (V total), the pore diameters (DBJH), and the wall thickness of the samples were inspected at 77 Kelvin for 6 h. The results indicate that according to the IUPAC classification of adsorption isotherms^60^, the N_2_ isotherm resembles the type III (Fig. 13). The obtained results of BET measurements were represented in Table 2. According to the obtained data, the surface area of the catalyst is 35.6 m^2^ g^−1^, which can provide a sufficient surface area for the catalyst to perform the desired synthesis.

**Table 2:** Results from the BET measurements of the FSiPSS nano-catalyst.

Parameter	Value
a_s_ (m^2 ^g^−1^)	3.56
V_m_ (cm^3 ^g^−1^)	8.17
Total pore volume	4.11
Mean pore diameter	462.19

**Figure 14:**
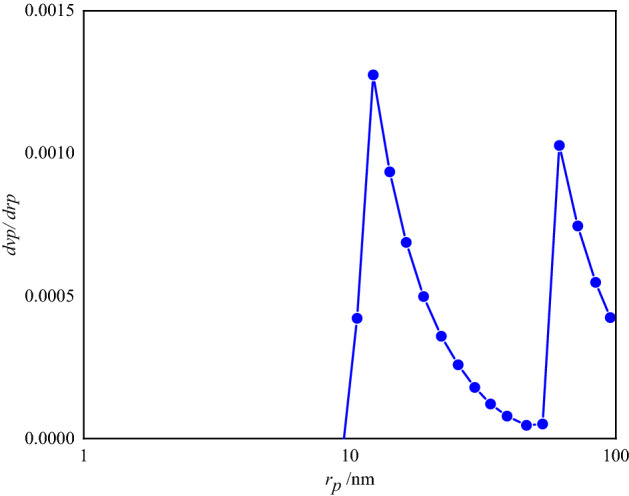
The BJH adsorption curve of the FSiPSS nano-catalyst.

Figure 14 shows the BJH adsorption curve of the FSiPSS nano-catalyst, which determined a pore size of approximately 12.24 nm.

### Optimization of the reaction conditions

After synthesis and full characterization of the novel FSiPSS nano-catalyst, the catalytic performance of the prepared nanomagnetic catalyst was also evaluated in the synthesis of pyranopyrazole derivatives. To attain this target, the reaction of ethyl acetoacetate, hydrazine hydrate, malononitrile, and benzaldehyde in the presence of the FSiPSS nano-catalyst was selected as a model reaction to find the best reaction conditions. The resulting data in various temperatures, amounts of the catalyst, and solvents are outlined in Table 3. The obtained data indicate that the best results were achieved when the reaction is carried out in the presence of 20 mg of the FSiPSS nano-catalyst in ethanol at 40 °C under ultrasonic conditions (entry 2). In addition, the effect of ultrasonication was also studied and its significance can be observed in time of the reaction which is drastically reduced as demonstrated in Tables 3 and 4.

**Table 3:** Optimization of the reaction conditions for the synthesis of pyranopyrazoles.

Entry	Solvent	Condition	Catal. amount (mg)	Time (min)	Yield (%)
1	C_2_H_5_OH	Sonication (50 Hz)/r.t	20	5	40
**2**	**C**_**2**_**H**_**5**_**OH**	**Sonication (50 Hz)/40 °C**	**20**	**3**	**80**
3	C_2_H_5_OH	Sonication (50 Hz)/50 °C	20	1	59
4	C_2_H_5_OH	Sonication (50 Hz)/60 °C	20	Fast	59
5	H_2_O	Sonication (50 Hz)/40 °C	20	Fast	45
6	CH_3_OH	Sonication (50 Hz)/40 °C	20	5	40
7	H_2_O/C_2_H_5_OH	Sonication (50 Hz)/40 °C	20	2	31
8	Ethyl acetate	Sonication (50 Hz)/40 °C	20	3	35
9	*n*-Hexane	Sonication (50 Hz)/40 °C	20	–	–
10	CH_3_Cl	Sonication (50 Hz)/40 °C	20	20	Trace
11	CH_3_CN	Sonication (50 Hz)/40 °C	20	2	Trace
12	C_2_H_5_OH	Sonication (50 Hz)/40 °C	–	–	–
13	C_2_H_5_OH	Sonication (50 Hz)/40 °C	10	3	40
14	C_2_H_5_OH	Sonication (50 Hz)/40 °C	30	3	40
15	C_2_H_5_OH	Reflux	20	30	70

Significant values are in bold.

**Table 4:** Study of the effect of ultrasonication and the role of each part of the catalyst in the reaction.

Entry	Catalyst	Condition	Time (min.)	Yield (%)	References
1	TEA-Im-IL-Cu	r.t	65	80	^30^
2	TrCl	Solvent-free, 60 °C	60	60	^41^
3	Cinchona alkaloid cupreine	CH_2_Cl_2_, r.t	27 h	92	^46^
4	FSi	Sonication/EtOH/40 °C	20	33	This work
5	FSiPS	Sonication/EtOH/40 °C	15	47	This work
6	FSiPSS	Sonication/EtOH/40 °C	3	80	This work

On other hand, the role of each part of the catalyst in the reaction was also investigated which the last step is the most effective according to entry 7 of Table 4.

### Synthesis of diverse pyranopyrazoles 5(a-n)

In the next step, with optimal reaction conditions in hand, the scope and generality of the presented method were investigated by examining the reaction of ethyl acetoacetate **1**, hydrazine hydrate **2**, various aromatic aldehydes **3(a-n),** and malononitrile **4a** or ethyl cyanoacetate **4b** in the presence of the catalytic amount of the FSiPSS nano-catalyst under ultrasonic conditions (Table 5).

**Table 5:**
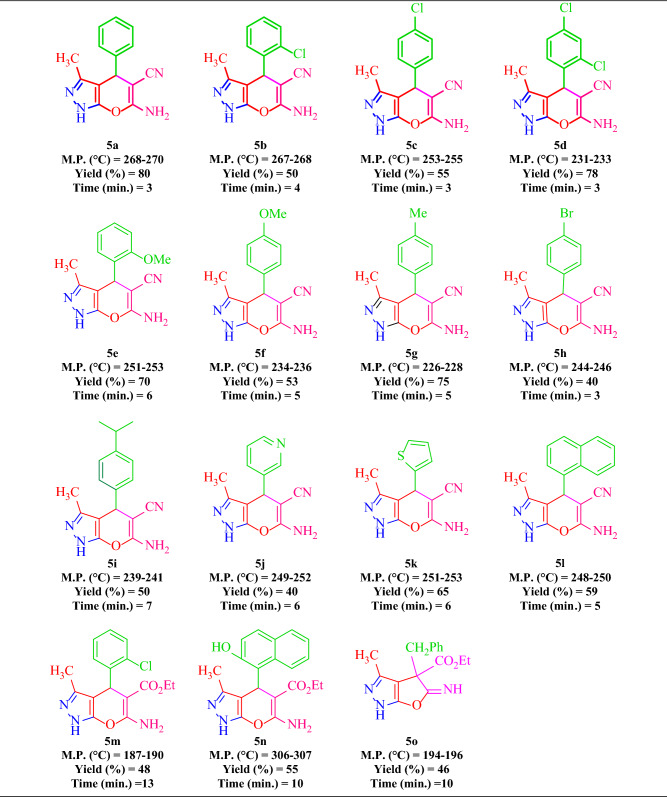
Synthesis of pyranopyrazoles **5(a-n)** by application of the FSiPSS nano-catalyst.

Reaction conditions: ethyl acetoacetate (1.0 mmol), hydrazine (1.0 mmol), aldehyde (1.0 mmol), malononitrile or ethyl cyanoacetate (1.5 mmol) and the FSiPSS nano-catalyst (20 mg) in EtOH (3.0 mL) under ultrasonic conditions.

### Proposed mechanism for the synthesis of diverse pyranopyrazoles 5(a-n)

**Figure 15:**
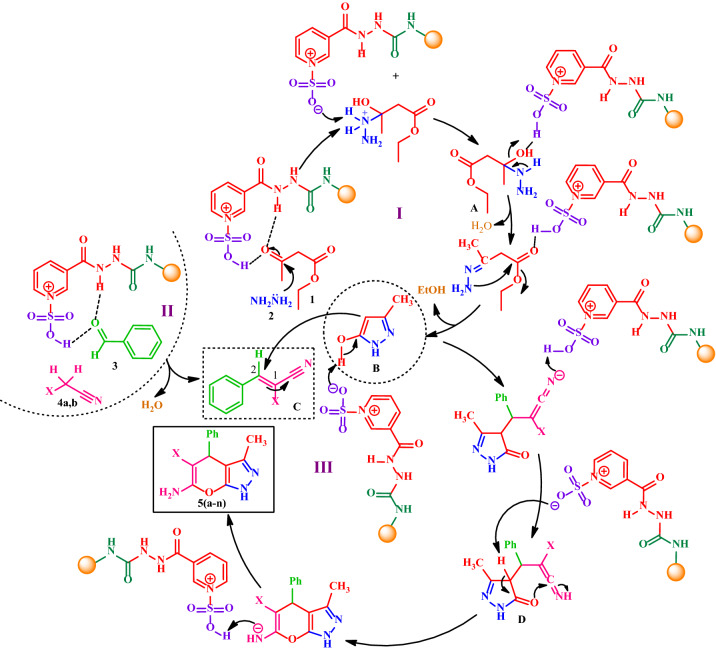
The proposed mechanism for the synthesis of diverse pyranopyrazoles **5(a-n)**.

A plausible mechanism for this reaction is depicted in Fig. 15. According to our suggested mechanism, firstly, a nucleophilic attack of hydrazine **2** to the carbonyl group of the activated ethyl acetoacetate **1** gives intermediate **A**, which subsequently with loss of H_2_O, and intramolecular nucleophilic attack of another NH_2_ group of hydrazine to the next carbonyl group of ethyl acetoacetate generates 3-methyl-1*H*-pyrazol-5-ol (intermediate **B**). In the next step, the benzylidene (intermediate **C**) containing the electron-poor C=C double bond is afforded by Knoevenagel condensation of the active methylene **4a,b** to the aromatic aldehydes **3**. Finally, the Michael addition of intermediate **B** to C-2 of the resulting intermediate **C** affords intermediate **D**, that after the loss of proton and intramolecular cyclization afford pyrano[2,3-*c*]pyrazoles **5(a-n)** in moderate to good yields (40–80%).

### Synthesis of ethyl 4-benzyl-5-imino-3-methyl-4,5-dihydro-1*H*-furo[2,3-*c*]pyrazole-4-carboxylate (5o) and the proposed mechanism

**Figure 16:**
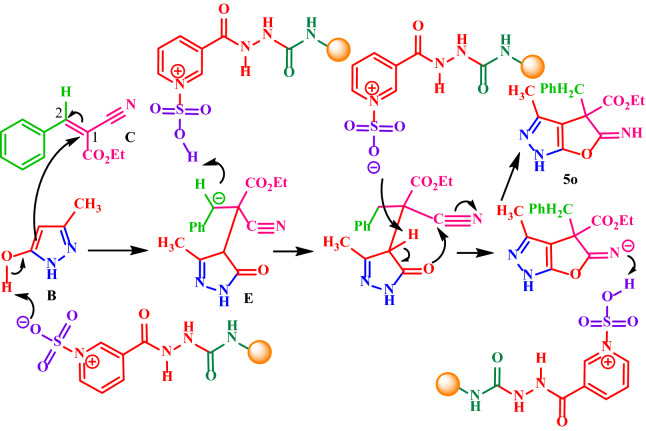
The proposed mechanism for the synthesis of the desired pyrazole **5o**.

Interestingly, the reaction of ethyl acetoacetate **1**, hydrazine **2**, and benzaldehyde **3a** with ethyl cyanoacetate **4b** under the same condition results in the formation of ethyl 4-benzyl-5-imino-3-methyl-4,5-dihydro-1*H*-furo[2,3-*c*]pyrazole-4-carboxylate **5o** in 46% yield. In this reaction, the obtained intermediate **B** adds to the C-1 of the resulting intermediate **C**, which led to the formation of the intermediate **E** as indicated in Fig. 16. Subsequently, an intramolecular nucleophilic attack of the oxygen of the carbonyl group to the cyano group gives the desired pyrazole **5o**.

### Reusability of the FSiPSS nano-catalyst

**Figure 17:**
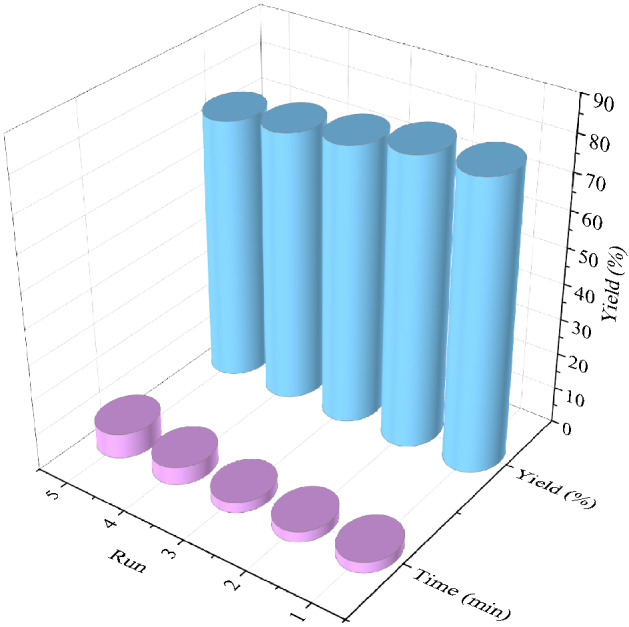
Reusability of the FSiPSS nano-catalyst.

In a separate study, recyclability and reusability of the magnetic FSiPSS nano-catalyst were tested for the synthesis of target molecule **5a** under optimal reaction conditions. At the end of each run, the magnetic FSiPSS nano-catalyst is separated from the reaction mixture by using a simple external magnet, washed thoroughly with EtOH, dried, and reused for the next run. Figure 17 demonstrates that the catalyst activity is preserved after four successive cycles without any considerable decrease in yield and reaction time.

### Comparison of the catalyst activities

In addition, the efficiency of our proposed protocol was also evaluated comparatively with some previously reported methods for the synthesis of pyranopyrazoles. According to Table 6, our proposed protocol used in this paper for the synthesis of pyranopyrazoles has no disadvantages and is accessible, applicable, and reusable with a very short reaction time, good for high yields and easy work-up.

**Table 6:** Comparison of the prepared catalyst with other reported catalysts.

	(1) TEA-Im-IL-Cu, rt, 65 min, 80%^30^
	(2) Isonicotinic acid (10 mol%), solvent-free, 85 °C, 10 min, 90%^32^
	(3) TrCl (10 mol%), solvent-free, 60 °C, 60 min, 80%^41^
	(4) Cinchona alkaloid cupreine (5 mol%), CH_2_Cl_2_, rt, 27 h, 92%^46^
	(5) The FSiPSS nano-catalyst (20 mg), EtOH, 40 °C, ultrasonic, fast, 80% [Current work]
	(1) TEA-Im-IL-Cu, rt, 25 min, 81%^30^
	(2) Isonicotinic acid (10 mol%), solvent-free, 85 °C, 25 min, 80%^32^
	(3) TrCl (10 mol%), solvent-free, 60 °C, 50 min, 83%^41^
	(4) The FSiPSS nano-catalyst (20 mg), EtOH, 40 °C, ultrasonic, fast, 70% [Current work]
	(1) Sodium ascorbate (15 mol%), EtOH:H_2_O (2:1), 50 °C, 15 min, 82%^48^
	(2) Meglumine (10 mol%), EtOH:H_2_O (9:1), rt, 17 min, 92%^49^
	(3) FSiPSS nano-catalyst (20 mg), EtOH, 40 °C, ultrasonic, fast, 78% [Current work]
	(1) Isonicotinic acid (10 mol%), solvent-free, 85 °C, 15 min, 85%^32^
	(2) Sodium ascorbate (15 mol%), EtOH:H_2_O (2:1), 50 °C, 12 min, 88%^48^
	(3) Meglumine (10 mol%), EtOH:H_2_O (9:1), rt, 12 min, 92%^49^
	(4) FSiPSS nano-catalyst (20 mg), EtOH, 40 °C, ultrasonic, fast, 65% [Current work]

## Conclusion

In summary, the synthesis of pyranopyrazole derivatives was performed using a sulfonic acid-functionalized nanomagnetic catalyst bearing the semicarbazide linkers as a new high-performance catalytic system under ultrasonic conditions. The simple and easy manufacturing procedure of this catalyst, along with the ability to recover and reuse it, makes it economical. The most attractive features of this procedure are low catalyst loading, short reaction times, good to high yields, lower temperature rather than previous works, compatibility with various functional groups, easy work-up, facile separation, and recyclability of catalyst.

Regarding the limitations of this project, the following points can be mentioned:

- Yield of the reaction: The yield of the reaction is between 40 and 80%, which can be improved in future by performing the reaction under new optimized conditions to increase the yield of the products.
- Replacement of malononitrile with ethyl cyanoacetate: among derivatives synthesized with ethyl cyanoacetate, only 3 derivatives were purified. By changing the reaction conditions, we hope to reduce the number of side reactions and improving the purification of the products resulting from the reaction with ethyl cyanoacetate.

## Supplementary Information

Supplementary Information.

## Data Availability

All data generated or analyzed during this study are included in the supplementary information file.
