# Supplementary material for: Design, preparation and application of the semicarbazide-pyridoyl-sulfonic acid-based nanocatalyst for the synthesis of pyranopyrazoles
Source: Sci Rep. 2022 Aug 23;12:14347. doi: 10.1038/s41598-022-18651-5 (PMC9399233; doi:10.1038/s41598-022-18651-5)
Supplement: Supplementary file 1 — Supplementary Information. [file 41598_2022_18651_MOESM1_ESM.docx]

**Supporting Information**

**(Scientific Reports)**

**Design, preparation and application of** **the semicarbazide-pyridoyl-sulfonic acid-based nanocatalyst for the synthesis of pyranopyrazoles**

**Masoumeh Beiranvand, Davood Habibi***

Department of Organic Chemistry, Faculty of Chemistry, Bu-Ali Sina University, Hamedan, Iran

*Corresponding author email: davood.habibi@gmail.com, Tel: +98 81 38380922; Fax: +98 81 38380709

Contents Pages

[**Spectra data** 1](#_Toc97457812)

[2-Nicotinoyl-N-(3-(triethoxysilyl)propyl)hydrazinecarboxamide (**Ligand A**) 1](#_Toc97457813)

[6-Amino-3-methyl-4-phenyl-1,4-dihydropyrano[2,3-*c*]pyrazole-5-carbonitrile (**5a**) 1](#_Toc97457814)

[6-Amino-4-(2-chlorophenyl)-3-methyl-1,4-dihydropyrano[2,3-*c*]pyrazole-5-carbonitrile (**5b**) 1](#_Toc97457815)

[6-Amino-4-(4-chlorophenyl)-3-methyl-1,4-dihydropyrano[2,3-*c*]pyrazole-5-carbonitrile (**5c**) 1](#_Toc97457816)

[6-Amino-4-(2,4-dichlorophenyl)-3-methyl-1,4-dihydropyrano[2,3-*c*]pyrazole-5-carbonitrile (**5d**) 1](#_Toc97457817)

[6-Amino-4-(2-methoxyphenyl)-3-methyl-1,4-dihydropyrano[2,3-*c*]pyrazole-5-carbonitrile (**5e**) 1](#_Toc97457818)

[6-Amino-4-(4-methoxyphenyl)-3-methyl-1,4-dihydropyrano[2,3-*c*]pyrazole-5-carbonitrile (**5f**) 2](#_Toc97457819)

[6-Amino-3-methyl-4-(p-tolyl)-1,4-dihydropyrano[2,3-*c*]pyrazole-5-carbonitrile (**5g**) 2](#_Toc97457820)

[6-Amino-4-(4-bromophenyl)-3-methyl-1,4-dihydropyrano[2,3-*c*]pyrazole-5-carbonitrile (**5h**) 2](#_Toc97457821)

[6-Amino-4-(4-isopropylphenyl)-3-methyl-1,4-dihydropyrano[2,3-*c*]pyrazole-5-carbonitrile (**5i**) 2](#_Toc97457822)

[6-Amino-3-methyl-4-(pyridin-3-yl)-1,4-dihydropyrano[2,3-*c*]pyrazole-5-carbonitrile (**5j**) 2](#_Toc97457823)

[6-Amino-3-methyl-4-(thiophen-2-yl)-1,4-dihydropyrano[2,3-*c*]pyrazole-5-carbonitrile (**5k**) 2](#_Toc97457824)

[6-Amino-3-methyl-4-(naphthalen-1-yl)-1,4-dihydropyrano[2,3-*c*]pyrazole-5-carbonitrile (**5l**) 3](#_Toc97457825)

[Ethyl 6-amino-4-(2-chlorophenyl)-3-methyl-1,4-dihydropyrano[2,3-*c*]pyrazole-5-carboxylate (**5m**) 3](#_Toc97457826)

[Ethyl 6-amino-4-(2-hydroxynaphthalen-1-yl)-3-methyl-1,4-dihydropyrano[2,3-*c*] pyrazole-5-carboxylate (**5n**) 3](#_Toc97457827)

[Ethyl 4-benzyl-5-imino-3-methyl-4,5-dihydro-1H-furo[2,3-*c*]pyrazole-4-carboxylate (**5o**) 3](#_Toc97457828)

[FT-IR Spectrum of 2-nicotinoyl-N-(3-(triethoxysilyl)propyl)hydrazinecarboxamide (**Ligand A**). 4](#_Toc97457829)

[HNMR Spectrum of 2-nicotinoyl-N-(3-(triethoxysilyl)propyl)hydrazinecarboxamide (**Ligand A**)*.* 4](#_Toc97457830)

[CNMR Spectrum of 2-nicotinoyl-N-(3-(triethoxysilyl)propyl)hydrazinecarboxamide (**Ligand A**). 5](#_Toc97457831)

[IR Spectrum of 6-amino-3-methyl-4-phenyl-1,4-dihydropyrano[2,3-*c*]pyrazole-5-carbonitrile (**5a**). 5](#_Toc97457832)

[HNMR Spectrum of 6-amino-3-methyl-4-phenyl-1,4-dihydropyrano[2,3-*c*]pyrazole-5-carbonitrile (**5a**). 6](#_Toc97457833)

[CNMR Spectrum of 6-amino-3-methyl-4-phenyl-1,4-dihydropyrano[2,3-*c*]pyrazole-5-carbonitrile (**5a**). 7](#_Toc97457834)

[IR Spectrum of 6-amino-4-(2-chlorophenyl)-3-methyl-1,4-dihydropyrano[2,3-*c*]pyrazole-5-carbonitrile (**5b**). 7](#_Toc97457835)

[HNMR Spectrum of 6-amino-4-(2-chlorophenyl)-3-methyl-1,4-dihydropyrano[2,3-*c*]pyrazole-5-carbonitrile (**5b**). 8](#_Toc97457836)

[IR Spectrum of 6-amino-4-(4-chlorophenyl)-3-methyl-1,4-dihydropyrano[2,3-*c*]pyrazole-5-carbonitrile (**5c**). 8](#_Toc97457837)

[HNMR Spectrum of 6-amino-4-(4-chlorophenyl)-3-methyl-1,4-dihydropyrano[2,3-*c*]pyrazole-5-carbonitrile (**5c**). 9](#_Toc97457838)

[IR Spectrum of 6-amino-4-(2,4-dichlorophenyl)-3-methyl-1,4-dihydropyrano[2,3-*c*]pyrazole-5-carbonitrile (**5d**). 9](#_Toc97457839)

[HNMR Spectrum of 6-amino-4-(2,4-dichlorophenyl)-3-methyl-1,4-dihydropyrano[2,3-*c*]pyrazole-5-carbonitrile (**5d**). 10](#_Toc97457840)

[IR Spectrum of 6-amino-4-(2-methoxyphenyl)-3-methyl-1,4-dihydropyrano[2,3-*c*]pyrazole-5-carbonitrile (**5e**). 10](#_Toc97457841)

[HNMR Spectrum of 6-amino-4-(2-methoxyphenyl)-3-methyl-1,4-dihydropyrano[2,3-*c*]pyrazole-5-carbonitrile (**5e**). 11](#_Toc97457842)

[IR Spectrum of 6-amino-4-(4-methoxyphenyl)-3-methyl-1,4-dihydropyrano[2,3-*c*]pyrazole-5-carbonitrile (**5f**). 11](#_Toc97457843)

[H NMR Spectrum of 6-amino-4-(4-methoxyphenyl)-3-methyl-1,4-dihydropyrano[2,3-*c*]pyrazole-5-carbonitrile (**5f**). 12](#_Toc97457844)

[IR Spectrum of 6-amino-3-methyl-4-(p-tolyl)-1,4-dihydropyrano[2,3-*c*]pyrazole-5-carbonitrile (**5g**). 12](#_Toc97457845)

[HNMR Spectrum of 6-amino-3-methyl-4-(p-tolyl)-1,4-dihydropyrano[2,3-*c*]pyrazole-5-carbonitrile (**5g**). 13](#_Toc97457846)

[IR Spectrum of 6-amino-4-(4-bromophenyl)-3-methyl-1,4-dihydropyrano[2,3-*c*]pyrazole-5-carbonitrile (**5h**). 13](#_Toc97457847)

[HNMR Spectrum of 6-amino-4-(4-bromophenyl)-3-methyl-1,4-dihydropyrano[2,3-*c*]pyrazole-5-carbonitrile (**5h**). 14](#_Toc97457848)

[IR Spectrum of 6-amino-4-(4-isopropylphenyl)-3-methyl-1,4-dihydropyrano[2,3-*c*]pyrazole-5-carbonitrile (**5i**). 14](#_Toc97457849)

[HNMR Spectrum of 6-amino-4-(4-isopropylphenyl)-3-methyl-1,4-dihydropyrano[2,3-*c*]pyrazole-5-carbonitrile (**5i**). 15](#_Toc97457850)

[IR Spectrum of 6-amino-3-methyl-4-(pyridin-3-yl)-1,4-dihydropyrano[2,3-c]pyrazole-5-carbonitrile (**5j**). 15](#_Toc97457851)

[HNMR Spectrum of 6-amino-3-methyl-4-(pyridin-3-yl)-1,4-dihydropyrano[2,3-*c*]pyrazole-5-carbonitrile (**5j**). 16](#_Toc97457852)

[IR Spectrum of 6-amino-3-methyl-4-(thiophen-2-yl)-1,4-dihydropyrano[2,3-*c*]pyrazole-5-carbonitrile (**5k**). 16](#_Toc97457853)

[HNMR Spectrum of 6-amino-3-methyl-4-(thiophen-2-yl)-1,4-dihydropyrano[2,3-*c*]pyrazole-5-carbonitrile (**5k**). 17](#_Toc97457854)

[IR Spectrum of 6-amino-3-methyl-4-(naphthalen-1-yl)-1,4-dihydropyrano[2,3-*c*]pyrazole-5-carbonitrile (**5l**). 17](#_Toc97457855)

[HNMR Spectrum of 6-amino-3-methyl-4-(naphthalen-1-yl)-1,4-dihydropyrano[2,3-*c*]pyrazole-5-carbonitrile (**5l**). 18](#_Toc97457856)

[IR Spectrum of ethyl 6-amino-4-(2-chlorophenyl)-3-methyl-1,4-dihydropyrano[2,3-*c*]pyrazole-5-carboxylate (**5m**). 18](#_Toc97457857)

[HNMR Spectrum of ethyl 6-amino-4-(2-chlorophenyl)-3-methyl-1,4-dihydropyrano[2,3-*c*]pyrazole-5-carboxylate (**5m**). 19](#_Toc97457858)

[CNMR Spectrum of ethyl 6-amino-4-(2-chlorophenyl)-3-methyl-1,4-dihydropyrano[2,3-*c*]pyrazole-5-carboxylate (**5m**). 20](#_Toc97457859)

[MS Spectrum of ethyl 6-amino-4-(2-chlorophenyl)-3-methyl-1,4-dihydropyrano[2,3-*c*]pyrazole-5-carboxylate (**5m**). 21](#_Toc97457860)

[IR Spectrum of ethyl 6-amino-4-(2-hydroxynaphthalen-1-yl)-3-methyl-1,4-dihydropyrano[2,3-*c*] pyrazole-5-carboxylate (**5n**). 22](#_Toc97457861)

[HNMR Spectrum of ethyl 6-amino-4-(2-hydroxynaphthalen-1-yl)-3-methyl-1,4-dihydropyrano[2,3-*c*] pyrazole-5-carboxylate (**5n**). 22](#_Toc97457862)

[CNMR Spectrum of ethyl 6-amino-4-(2-hydroxynaphthalen-1-yl)-3-methyl-1,4-dihydropyrano[2,3-*c*] pyrazole-5-carboxylate (**5n**). 23](#_Toc97457863)

[MS Spectrum of ethyl 6-amino-4-(2-hydroxynaphthalen-1-yl)-3-methyl-1,4-dihydropyrano[2,3-*c*] pyrazole-5-carboxylate (**5n**). 24](#_Toc97457864)

[IR Spectrum of ethyl 4-benzyl-5-imino-3-methyl-4,5-dihydro-1H-furo[2,3-*c*]pyrazole-4-carboxylate (**5o**). 25](#_Toc97457865)

[HNMR Spectrum of ethyl 4-benzyl-5-imino-3-methyl-4,5-dihydro-1H-furo[2,3-*c*]pyrazole-4-carboxylate (**5o**). 25](#_Toc97457866)

[CNMR Spectrum of ethyl 4-benzyl-5-imino-3-methyl-4,5-dihydro-1H-furo[2,3-*c*]pyrazole-4-carboxylate (**5o**). 26](#_Toc97457867)

[MS Spectrum of ethyl 4-benzyl-5-imino-3-methyl-4,5-dihydro-1H-furo[2,3-*c*]pyrazole-4-carboxylate (**5o**). 27](#_Toc97457868)

# **Spectra data**

*2-Nicotinoyl-N-(3-(triethoxysilyl)propyl)hydrazinecarboxamide* (**Ligand A**):

Pale yellow solid, M.p. 114-116 °C, IR (KBr, cm^-1^): 3323, 2974, 1644, 1596, and 1079. ^1^H NMR (250 MHz, DMSO) δ 10.29 (s, 1H), 9.01 (s, 1H), 8.69 (d, *J* = 4.8 Hz, 1H), 8.19 (d, *J* = 8.1 Hz, 1H), 7.88 (s, 1H), 7.49 (dd, *J* = 8.0, 4.8 Hz, 1H), 6.59 (t, *J* = 5.9 Hz, 1H), 3.70 (q, *J* = 7.0 Hz, 6H), 2.97 (q, *J* = 6.8 Hz, 2H), 1.42 (t, *J* = 8.3 Hz, 2H), 1.10 (t, *J* = 7.0 Hz, 9H), 0.49 (t, *J* = 8.4 Hz, 2H). ^13^C NMR (62.5 MHz, DMSO-*d_6_*): δ 165.37, 158.61, 152.60, 149.05, 135.66, 128.83, 123.82, 58.08, 42.36, 39.88, 23.78, 18.58, 7.57.

*6-Amino-3-methyl-4-phenyl-1,4-dihydropyrano[2,3-c]pyrazole-5-carbonitrile* (**5a**):

Pale yellow solid, M.p. 268-270 °C, IR (KBr, cm^-1^): 3372, 3170, 2192, 1649, 1611, and 1044. ^1^H NMR (250 MHz, DMSO-*d_6_*): δ 12.09 (s, 1H), 7.29 (t, *J* = 7.2 Hz, 2H), 7.21 (d, *J* = 6.5 Hz, 1H), 7.15 (d, *J* = 7.5 Hz, 2H), 6.87 (s, 2H), 4.57 (s, 1H), 1.76 (s, 3H). ^13^C NMR (62.5 MHz, DMSO-*d_6_*): δ 161.32, 155.21, 144.88, 136.04, 128.88, 127.91, 127.18, 121.25, 98.08, 57.63, 39.90, 36.67, 10.17.

*6-Amino-4-(2-chlorophenyl)-3-methyl-1,4-dihydropyrano[2,3-c]pyrazole-5-carbonitrile* (**5b**):

White solid, M.p. 268-268 °C, IR (KBr, cm^-1^): 3390, 3165, 2190, 1654, 1610 and 1053. ^1^H NMR (250 MHz, DMSO) δ 12.11 (s, 1H), 7.38 (d, *J* = 7.7 Hz, 1H), 7.25 (t, *J* = 8.1 Hz, 2H), 7.16 (d, *J* = 7.7 Hz, 1H), 6.93 (s, 2H), 5.04 (s, 1H), 1.74 (s, 3H).

*6-Amino-4-(4-chlorophenyl)-3-methyl-1,4-dihydropyrano[2,3-c]pyrazole-5-carbonitrile* (**5c**):

White solid, M.p. 253-255 °C, IR (KBr, cm^-1^): 3478, 3232, 2193, 1648, 1596 and 1052. ^1^H NMR (250 MHz, DMSO-*d_6_*): δ 12.11 (s, 1H), 7.35 (d, *J* = 8.0 Hz, 2H), 7.17 (d, *J* = 8.1 Hz, 2H), 6.90 (s, 2H, CH), 4.61 (s, 1H), 1.76 (s, 3H).

*6-Amino-4-(2,4-dichlorophenyl)-3-methyl-1,4-dihydropyrano[2,3-c]pyrazole-5-carbonitrile* (**5d**):

White solid, M.p. 231-233 °C, IR (KBr, cm^-1^): 3485, 3251, 2185, 1642, 1592 and 1058. ^1^H NMR (250 MHz, DMSO) δ 12.14 (s, 1H), 7.54 (s, 1H), 7.37 (d, *J* = 8.4 Hz, 1H), 7.18 (d, *J* = 8.5 Hz, 1H), 6.99 (s, 2H), 5.03 (s, 1H), 1.75 (s, 3H).

*6-Amino-4-(2-methoxyphenyl)-3-methyl-1,4-dihydropyrano[2,3-c]pyrazole-5-carbonitrile* (**5e**):

Pale yellow solid, M.p. 251-253 °C, IR (KBr, cm^-1^): 3376, 3159, 2839, 2195, 1656, 1613, 1488 and 1028. ^1^H NMR (250 MHz, DMSO) δ 11.95 (s, 1H), 7.15 (t, *J* = 7.7 Hz, 1H), 6.97 (d, *J* = 7.9 Hz, 2H), 6.88 (t, *J* = 7.3 Hz, 1H), 6.71 (s, 2H), 4.95 (s, 1H), 3.76 (s, 3H), 1.77 (s, 3H).

*6-Amino-4-(4-methoxyphenyl)-3-methyl-1,4-dihydropyrano[2,3-c]pyrazole-5-carbonitrile* (**5f**):

White solid, M.p. 234-236 °C, IR (KBr, cm^-1^): 3485, 3254, 2064, 2192, 1643, 1598, 1492 and 1030. ^1^H NMR (250 MHz, DMSO) δ 12.05 (s, 1H), 7.07 (d, *J* = 2.4 Hz, 1H), 7.03 (d, *J* = 2.3 Hz, 1H), 6.86 (s, 2H), 6.83 – 6.80 (m, 2H), 4.51 (s, 1H), 3.70 (s, 3H), 1.75 (s, 3H).

*6-Amino-3-methyl-4-(p-tolyl)-1,4-dihydropyrano[2,3-c]pyrazole-5-carbonitrile* (**5g**):

White solid, M.p. 226-228 °C, IR (KBr, cm^-1^): 3409, 3189, 2193, 1647, 1599 and 1044. ^1^H NMR (250 MHz, DMSO-*d_6_*): δ 12.06 (s, 1H), 7.10-7.00 (m, 4H), 6.82 (s, 2H), 4.51 (s, 1H), 1.75 (s, 3H).

*6-Amino-4-(4-bromophenyl)-3-methyl-1,4-dihydropyrano[2,3-c]pyrazole-5-carbonitrile* (**5h**):

White solid, M.p. 244-246 °C, IR (KBr, cm^-1^): 3483, 3230, 2190, 1642, 1595, 1490, 1402 and 1010. ^1^H NMR (250 MHz, DMSO) δ 12.12 (s, 1H), 7.47 (d, *J* = 8.0 Hz, 2H), 7.10 (d, *J* = 8.0 Hz, 2H), 6.91 (s, 2H), 4.58 (s, 1H), 1.76 (s, 3H).

*6-Amino-4-(4-isopropylphenyl)-3-methyl-1,4-dihydropyrano[2,3-c]pyrazole-5-carbonitrile* (**5i**):

White solid, M.p. 239-241 °C, IR (KBr, cm^-1^): 3494, 3233, 2961, 2196, 1613, 1597, 1490, 1398 and 1053. ^1^H NMR (250 MHz, DMSO) δ 12.06 (s, 1H), 7.16 (d, *J* = 7.9 Hz, 2H), 7.04 (d, *J* = 7.8 Hz, 2H), 6.83 (s, 2H), 4.52 (s, 1H), 2.81 (q, *J* = 6.9 Hz, 1H), 1.77 (s, 3H), 1.16 (d, *J* = 6.9 Hz, 6H).

*6-Amino-3-methyl-4-(pyridin-3-yl)-1,4-dihydropyrano[2,3-c]pyrazole-5-carbonitrile* (**5j**):

Cream solid, M.p. 249-252 °C, IR (KBr, cm^-1^): 3394, 3178, 2193, 1647, 1598, 1490, 1402 and 1034. ^1^H NMR (250 MHz, DMSO) δ 12.16 (s, 1H), 8.43 (d, *J* = 3.4 Hz, 2H), 7.51 (d, *J* = 7.9 Hz, 1H), 7.33 (dd, *J* = 7.9, 4.7 Hz, 1H), 6.98 (s, 2H), 4.68 (s, 1H), 1.77 (s, 3H).

*6-Amino-3-methyl-4-(thiophen-2-yl)-1,4-dihydropyrano[2,3-c]pyrazole-5-carbonitrile* (**5k**):

Orange solid, M.p. 251-253 °C, IR (KBr, cm^-1^): 3360, 3172, 2192, 1649, 1610, 1043. ^1^H NMR (250 MHz, DMSO-*d_6_*): δ 12.16 (s, 1H), 7.35 (d, *J* = 5.0 Hz, 1H), 6.98 (s, 2H), 6.93 (s, 2H), 4.97 (s, 1H), 1.89 (s, 3H).

*6-Amino-3-methyl-4-(naphthalen-1-yl)-1,4-dihydropyrano[2,3-c]pyrazole-5-carbonitrile* (**5l**):

Cream solid, M.p. 248-250 °C, IR (KBr, cm^-1^): 3373, 3153, 2190, 1652, 1598, 1489, 1404 and 1045. ^1^H NMR (250 MHz, DMSO) δ 12.12 (s, 1H), 7.92 – 7.80 (m, 3H), 7.75 (s, 1H), 7.50 – 7.40 (m, 2H), 7.23 (d, *J* = 8.3 Hz, 1H), 6.95 (s, 2H), 4.77 (s, 1H), 1.73 (s, 3H).

*Ethyl 6-amino-4-(2-chlorophenyl)-3-methyl-1,4-dihydropyrano[2,3-c]pyrazole-5-carboxylate* (**5m**):

Cream solid, M.p. 187-190 °C, IR (KBr, cm^-1^): 3391, 3292, 1745, 1668, 1483 and 1101. ^1^H NMR (250 MHz, DMSO-*d_6_*): δ 11.99 (s, 1H), 7.82 (s, 2H), 7.29 (d, *J* = 7.7 Hz, 2H), 7.17-7.07 (m, 2H), 5.21 (s, 1H), 3.79 (q, *J* = 7.1 Hz, 2H), 1.88 (s, 3H), 0.90 (t, 3H). ^13^C NMR (62.5 MHz, DMSO-*d_6_*): δ 169.22, 162.51, 154.94, 145.05, 135.81, 132.00, 130.48, 129.16, 127.71, 99.67, 76.31, 62.61, 58.98, 39.93, 32.25, 14.30, 10.17. MS: ESI-mass: m/z = 333.

*Ethyl 6-amino-4-(2-hydroxynaphthalen-1-yl)-3-methyl-1,4-dihydropyrano[2,3-c] pyrazole-5-carboxylate* (**5n**):

Yellow solid, M.p. 306-307 °C, IR (KBr, cm^-1^): 3063, 1733, 1566, 1214. ^1^H NMR (250 MHz, DMSO-*d_6_*): δ 9.72 (s, 1H), 8.58 (d, 1H), 8.33 (d, 1H), 8.06 (d, 1H), 7.76 (d, 1H), 7.61 (dd, *J* = 4.8 Hz, 2H), 7.43 (s, 2H), 7.20 (s, 1H), 5.25 (s, 1H), 4.03 (t, 2H), 2.00 (s, 3H), 1.18 (t, 3H). ^13^C NMR (62.5 MHz, DMSO-*d_6_*): δ 169.17, 160.86, 155.45, 150.00, 137.60, 130.25, 129.81, 129.50, 127.29, 124.84, 122.96, 117.17, 115.45, 112.74, 101.21, 59.08, 25.22, 14.88, 10.35. MS: ESI-mass: m/z = 365.

*Ethyl 4-benzyl-5-imino-3-methyl-4,5-dihydro-1H-furo[2,3-c]pyrazole-4-carboxylate* (**5o**):

Cream solid, 194-196 °C, IR (KBr, cm^-1^): 3339, 1744, 1661 and 1307. ^1^H NMR (250 MHz, DMSO-*d_6_*) δ 10.77 (s, 1H), 7.78-6.86 (m, 6H), 4.76 (d, *J* = 11.2 Hz, 1H), 4.29 (d, *J* = 10.3 Hz, 1H), 4.09-3.85 (m, 2H), 2.00 (d, *J* = 19.0 Hz, 3H), 0.93 (d, *J* = 31.2 Hz, 3H). ^13^C NMR (62.5 MHz, DMSO-*d_6_*) δ 165.89, 159.54, 140.96, 137.91, 137.65, 128.86, 128.22, 127.94, 127.50, 117.33, 100.10, 99.56, 62.42, 42.41, 39.87, 13.87, 10.34. MS: ESI-mass: m/z = 299.


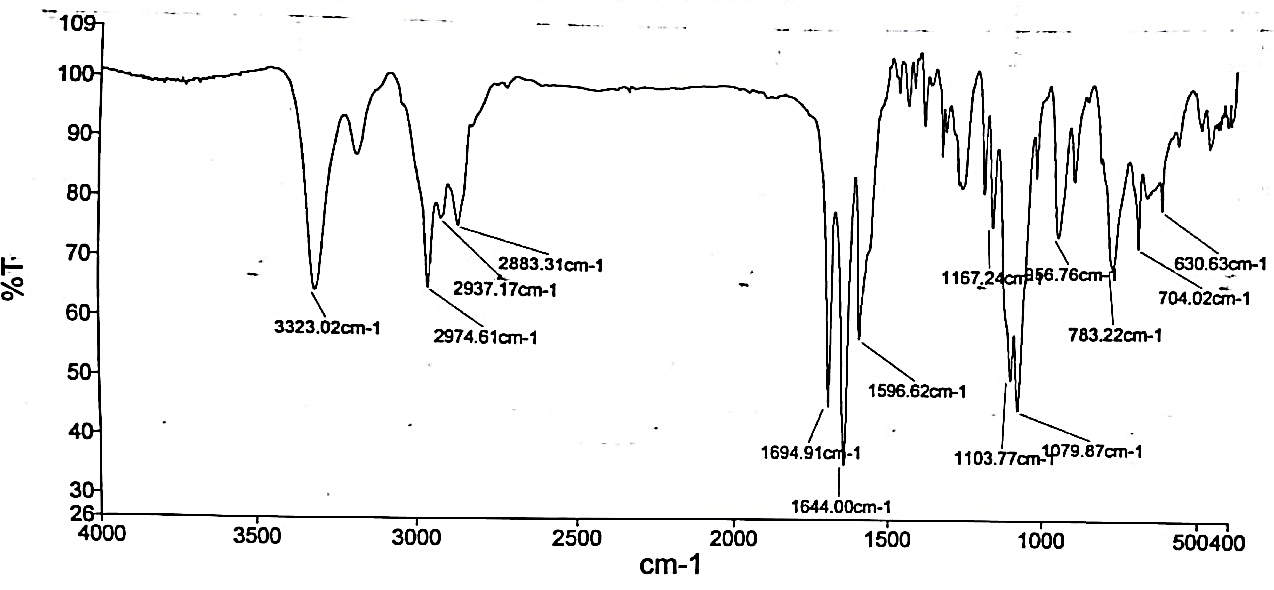


## FT-IR Spectrum of *2-nicotinoyl-N-(3-(triethoxysilyl)propyl)hydrazinecarboxamide* (**Ligand A**).

**
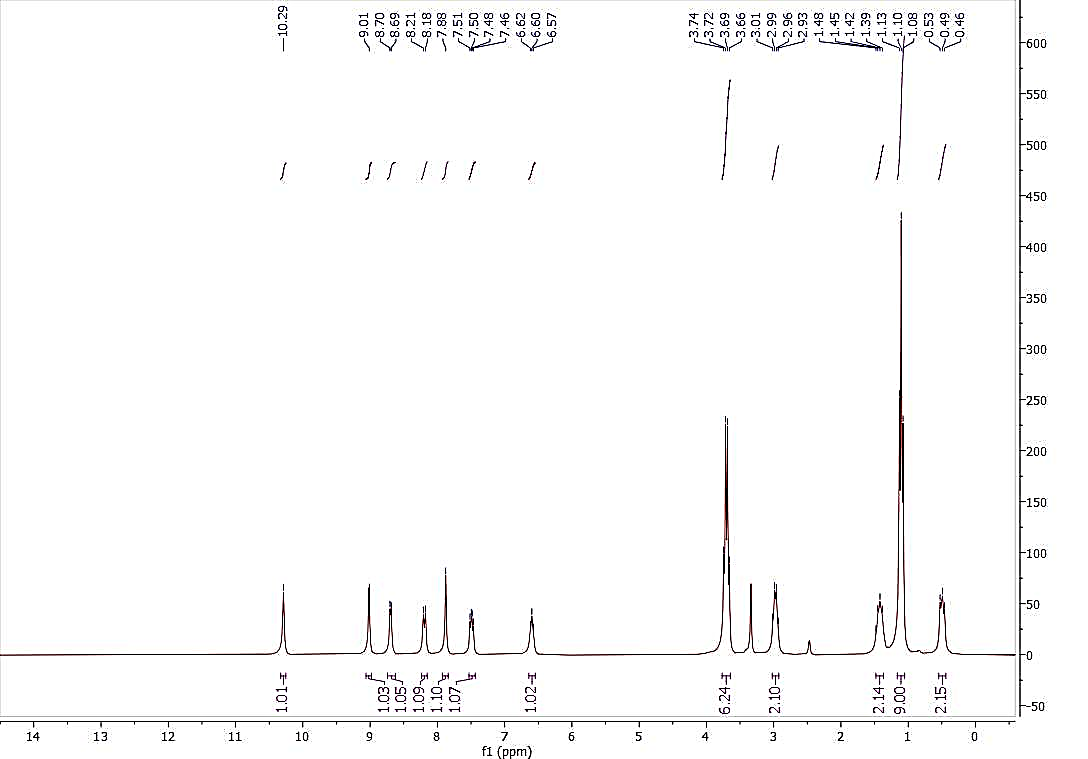
**

## HNMR Spectrum of *2-nicotinoyl-N-(3-(triethoxysilyl)propyl)hydrazinecarboxamide* (**Ligand A**).

**
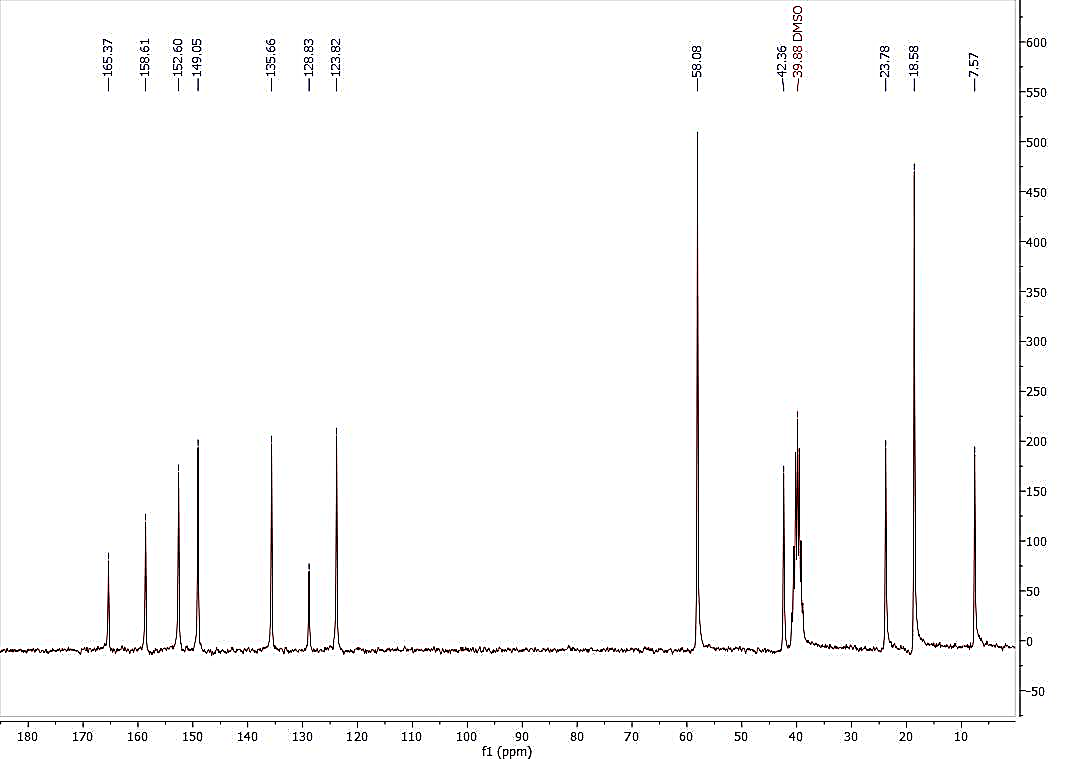
**

## CNMR Spectrum of *2-nicotinoyl-N-(3-(triethoxysilyl)propyl)hydrazinecarboxamide* (**Ligand A**).


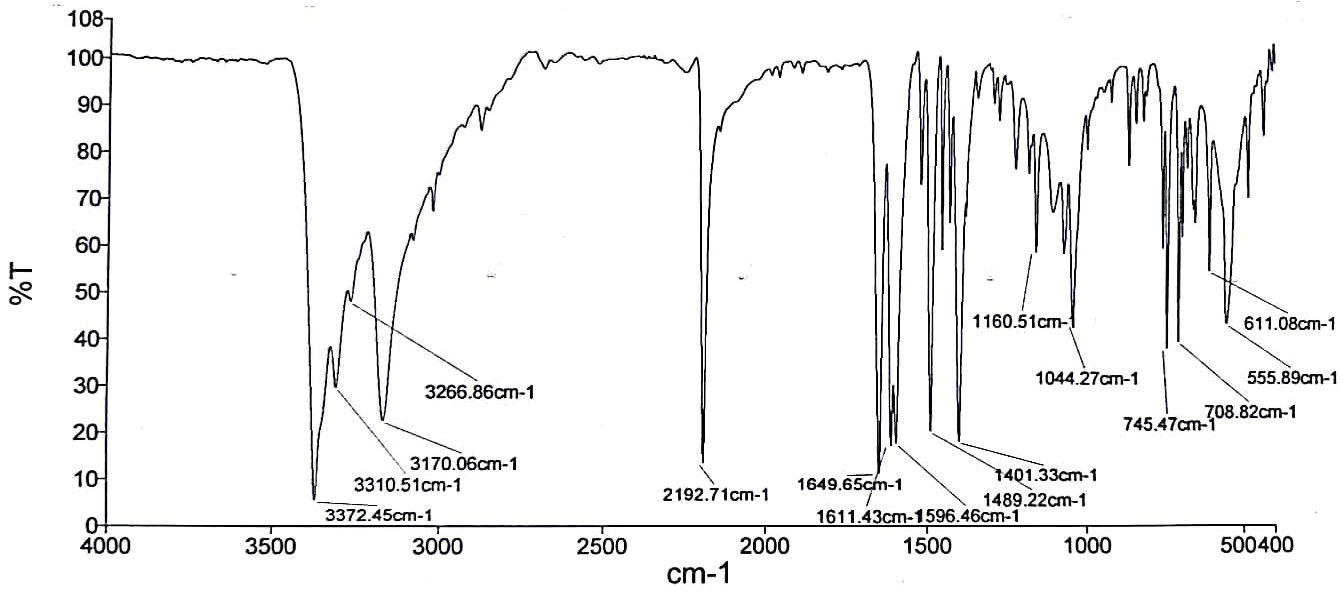


## IR Spectrum of *6-amino-3-methyl-4-phenyl-1,4-dihydropyrano[2,3-c]pyrazole-5-carbonitrile* (**5a**).


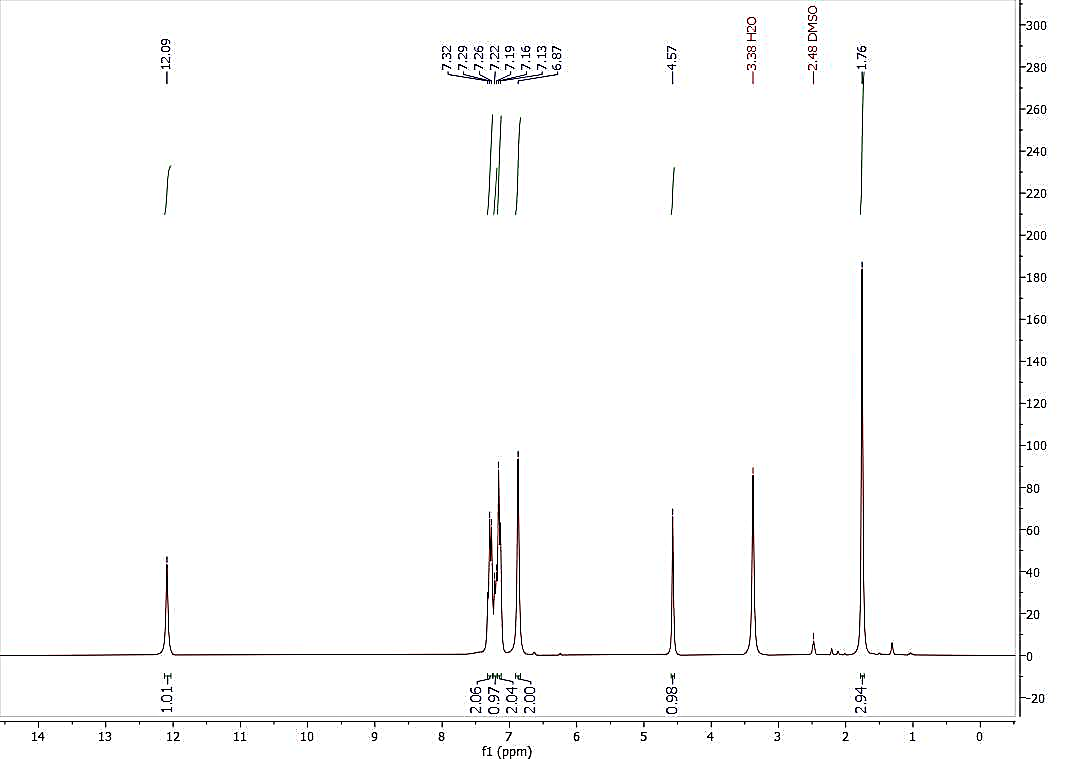


## HNMR Spectrum of *6-amino-3-methyl-4-phenyl-1,4-dihydropyrano[2,3-c]pyrazole-5-carbonitrile* (**5a**).


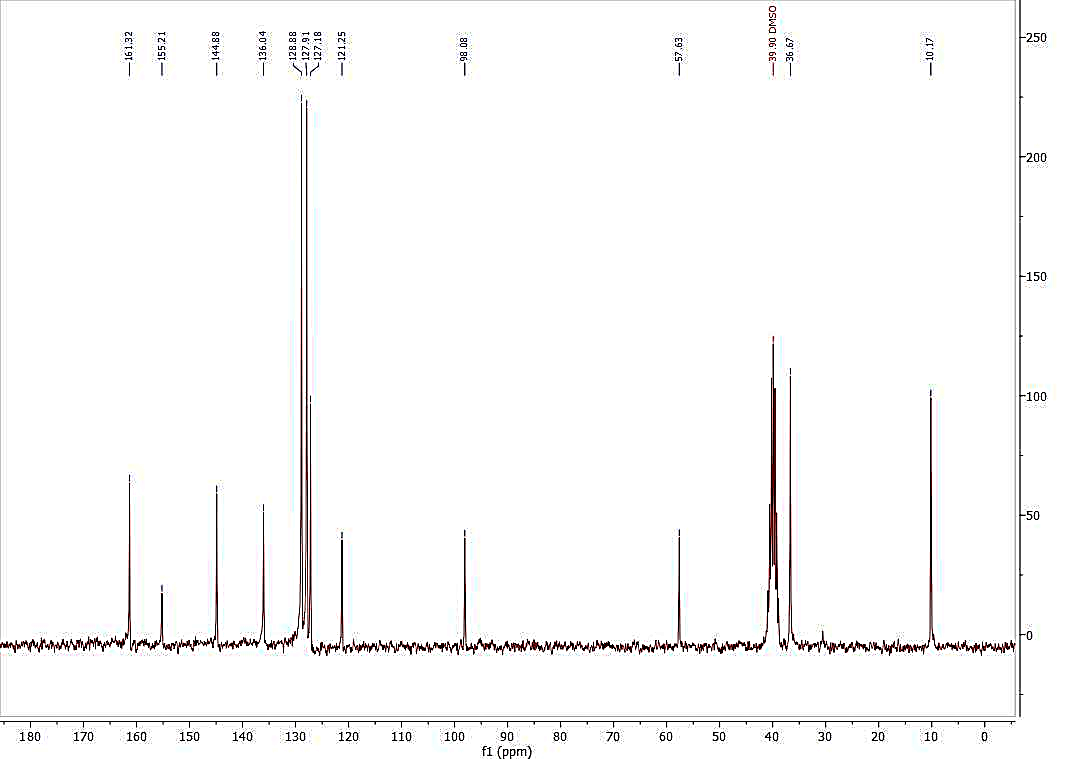


## CNMR Spectrum of *6-amino-3-methyl-4-phenyl-1,4-dihydropyrano[2,3-c]pyrazole-5-carbonitrile* (**5a**).


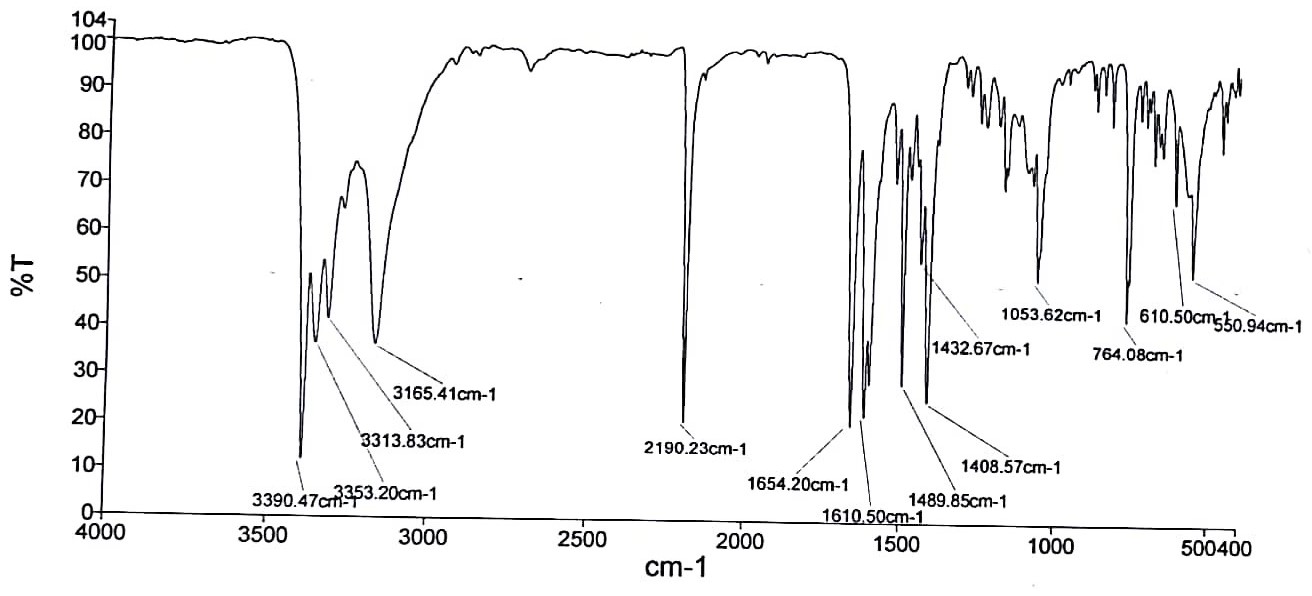


## IR Spectrum of *6-amino-4-(2-chlorophenyl)-3-methyl-1,4-dihydropyrano[2,3-c]pyrazole-5-carbonitrile* (**5b**).


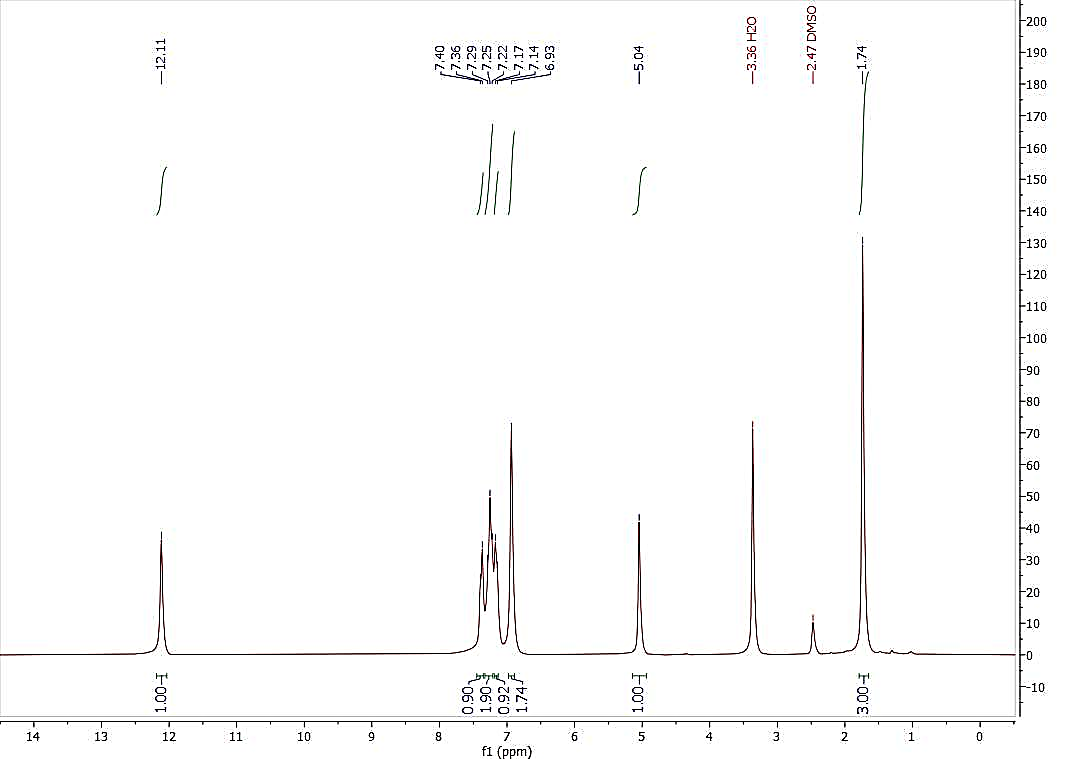


## HNMR Spectrum of *6-amino-4-(2-chlorophenyl)-3-methyl-1,4-dihydropyrano[2,3-c]pyrazole-5-carbonitrile* (**5b**).


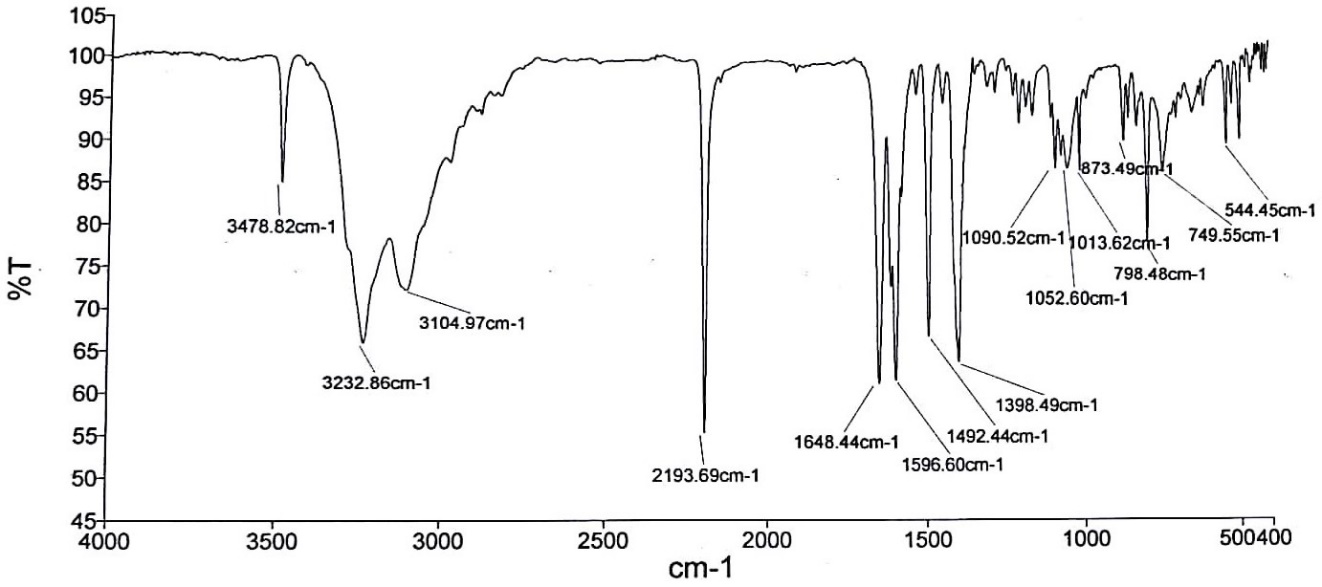


## IR Spectrum of *6-amino-4-(4-chlorophenyl)-3-methyl-1,4-dihydropyrano[2,3-c]pyrazole-5-carbonitrile* (**5c**).


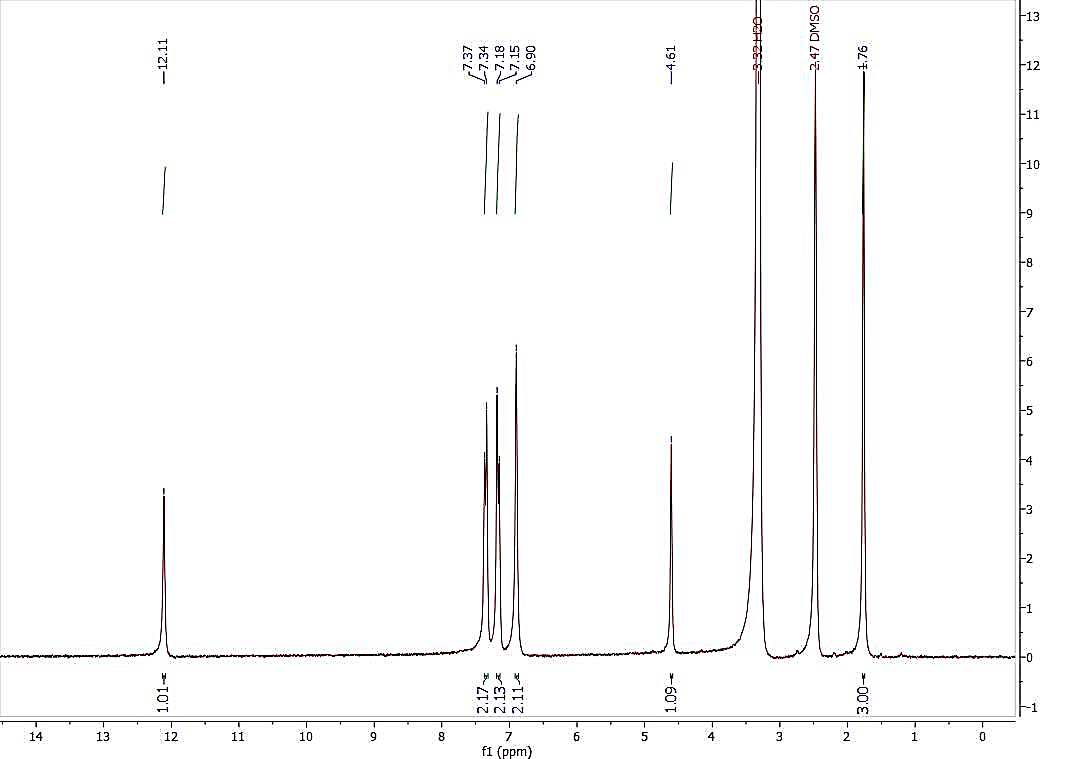


## HNMR Spectrum of *6-amino-4-(4-chlorophenyl)-3-methyl-1,4-dihydropyrano[2,3-c]pyrazole-5-carbonitrile* (**5c**).

**
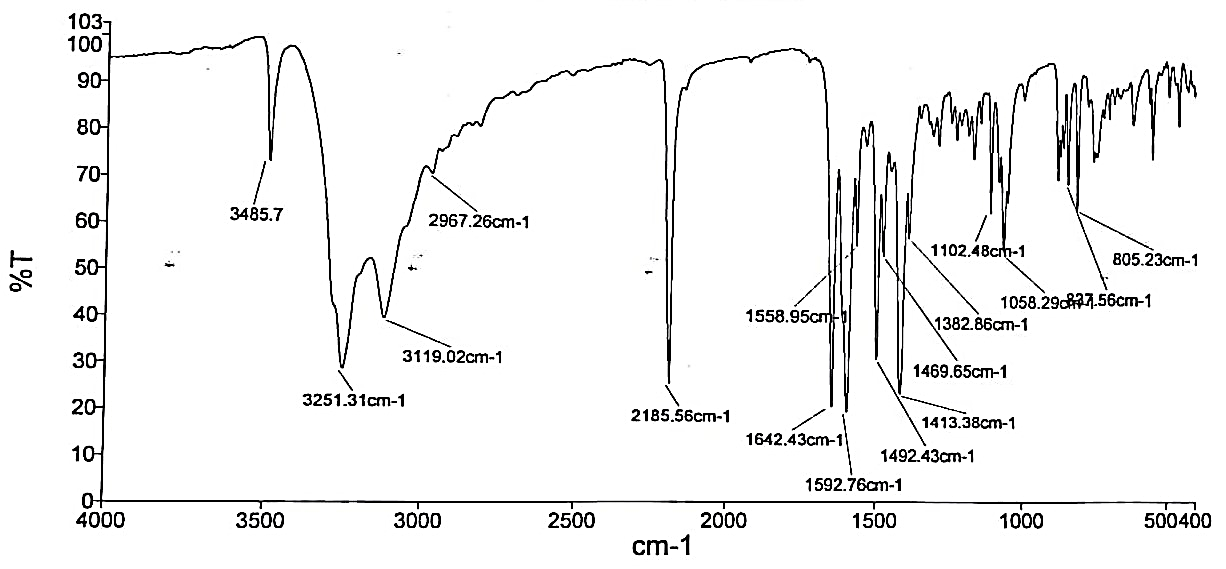
**

## IR Spectrum of *6-amino-4-(2,4-dichlorophenyl)-3-methyl-1,4-dihydropyrano[2,3-c]pyrazole-5-carbonitrile* (**5d**).

**
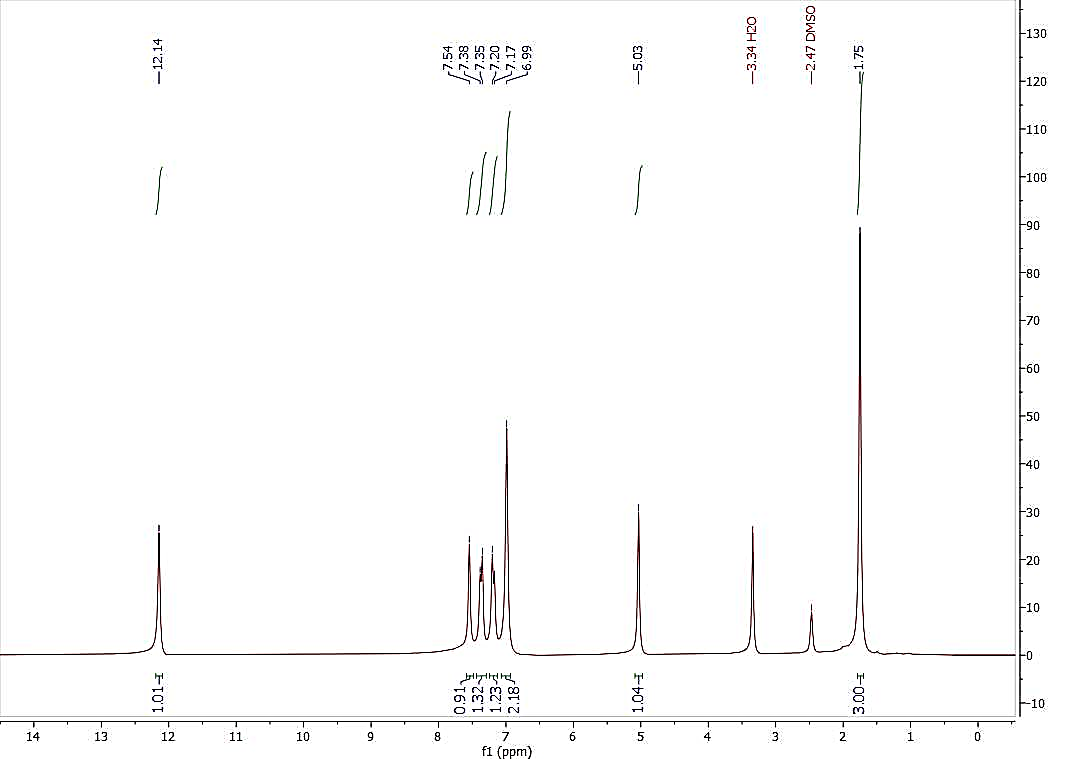
**

## HNMR Spectrum of *6-amino-4-(2,4-dichlorophenyl)-3-methyl-1,4-dihydropyrano[2,3-c]pyrazole-5-carbonitrile* (**5d**).

**
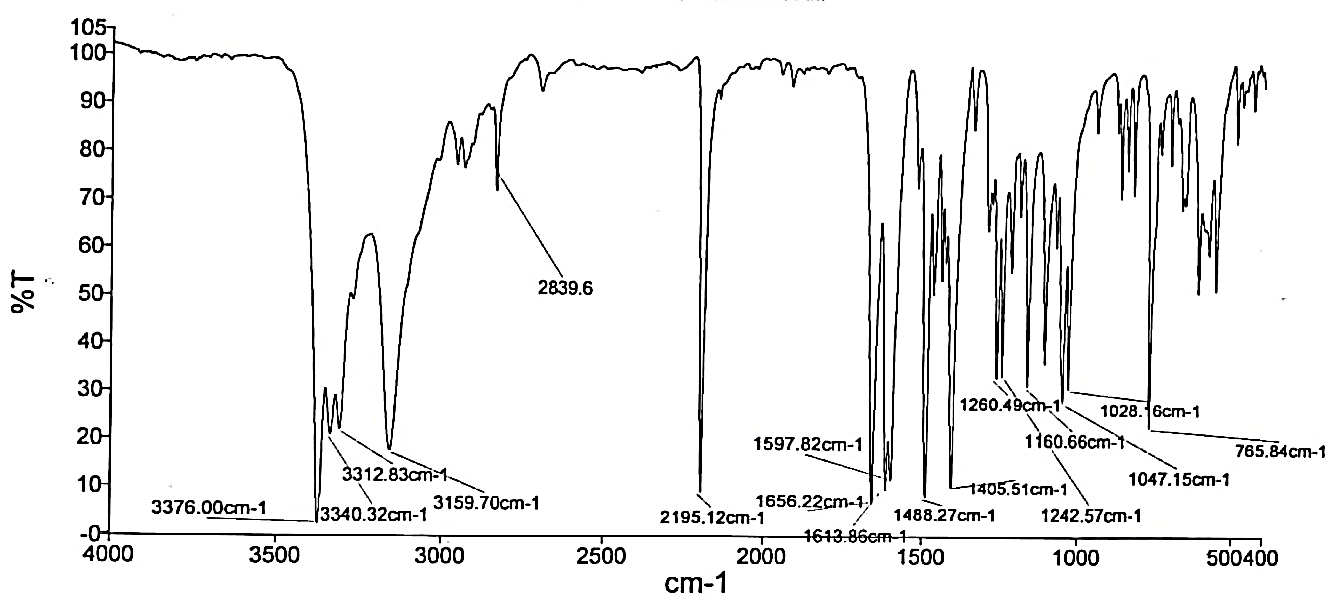
**

## IR Spectrum of *6-amino-4-(2-methoxyphenyl)-3-methyl-1,4-dihydropyrano[2,3-c]pyrazole-5-carbonitrile* (**5e**).

**
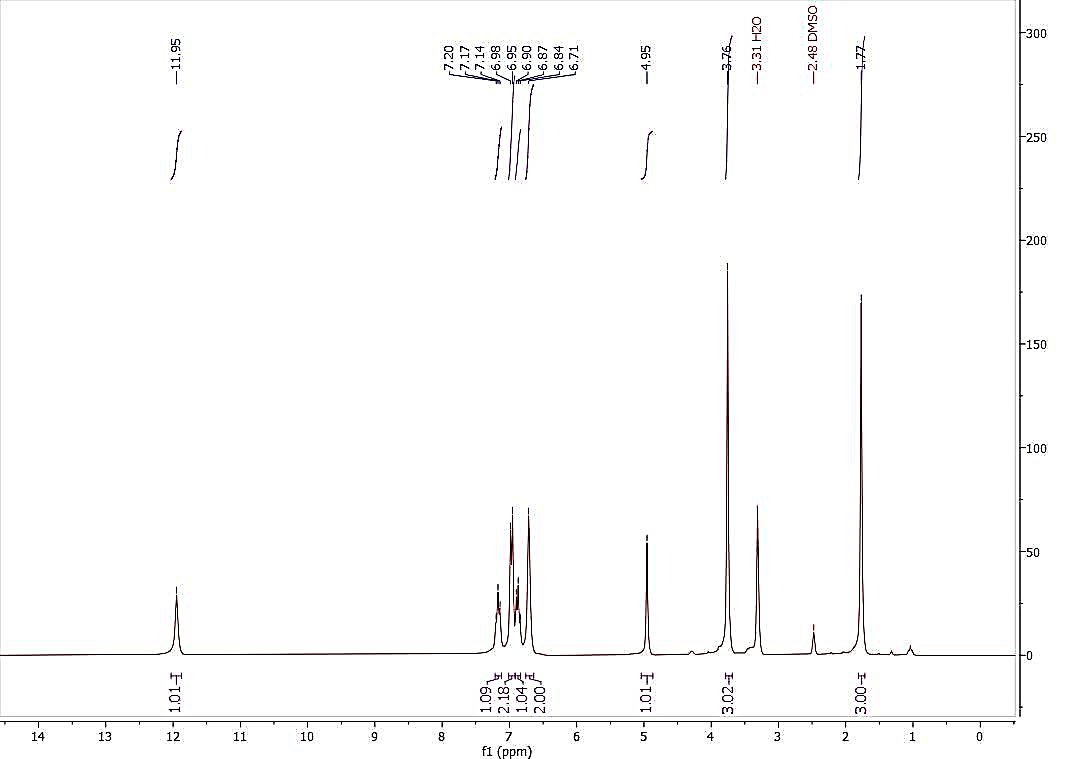
**

## HNMR Spectrum of *6-amino-4-(2-methoxyphenyl)-3-methyl-1,4-dihydropyrano[2,3-c]pyrazole-5-carbonitrile* (**5e**).

**
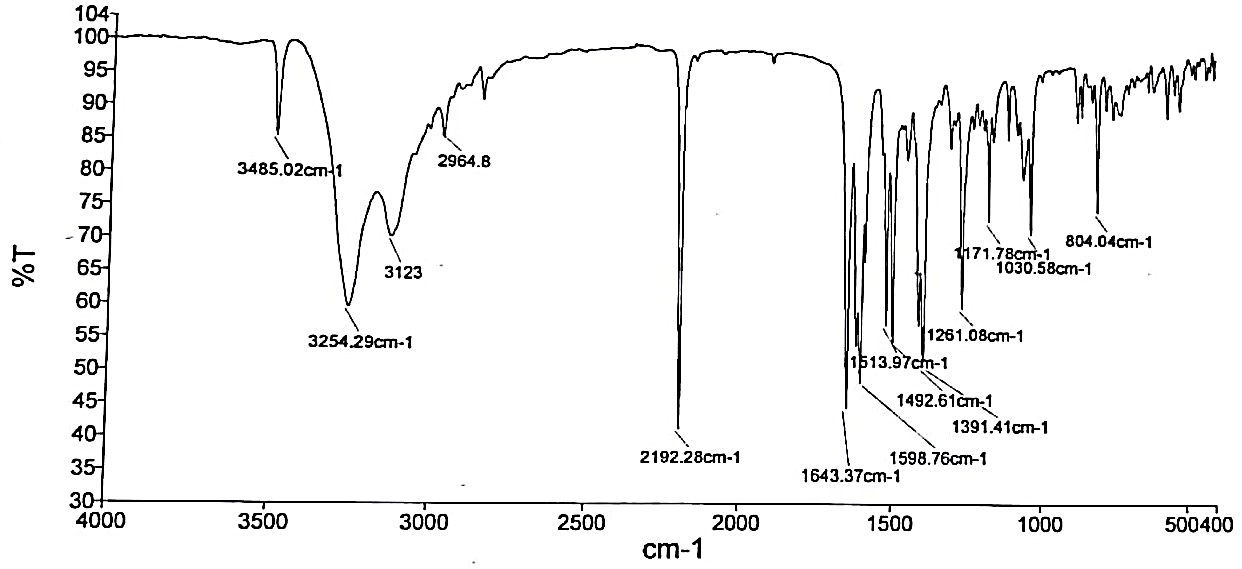
**

## IR Spectrum of *6-amino-4-(4-methoxyphenyl)-3-methyl-1,4-dihydropyrano[2,3-c]pyrazole-5-carbonitrile* (**5f**).

**
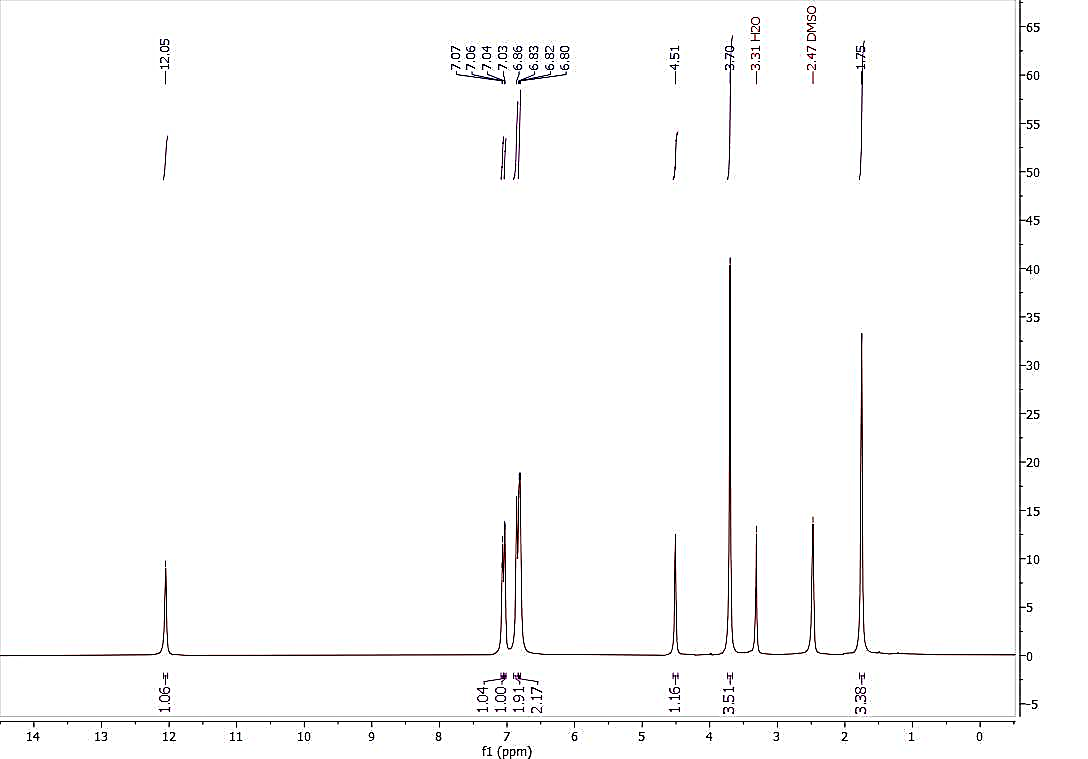
**

## H NMR Spectrum of *6-amino-4-(4-methoxyphenyl)-3-methyl-1,4-dihydropyrano[2,3-c]pyrazole-5-carbonitrile* (**5f**).

**
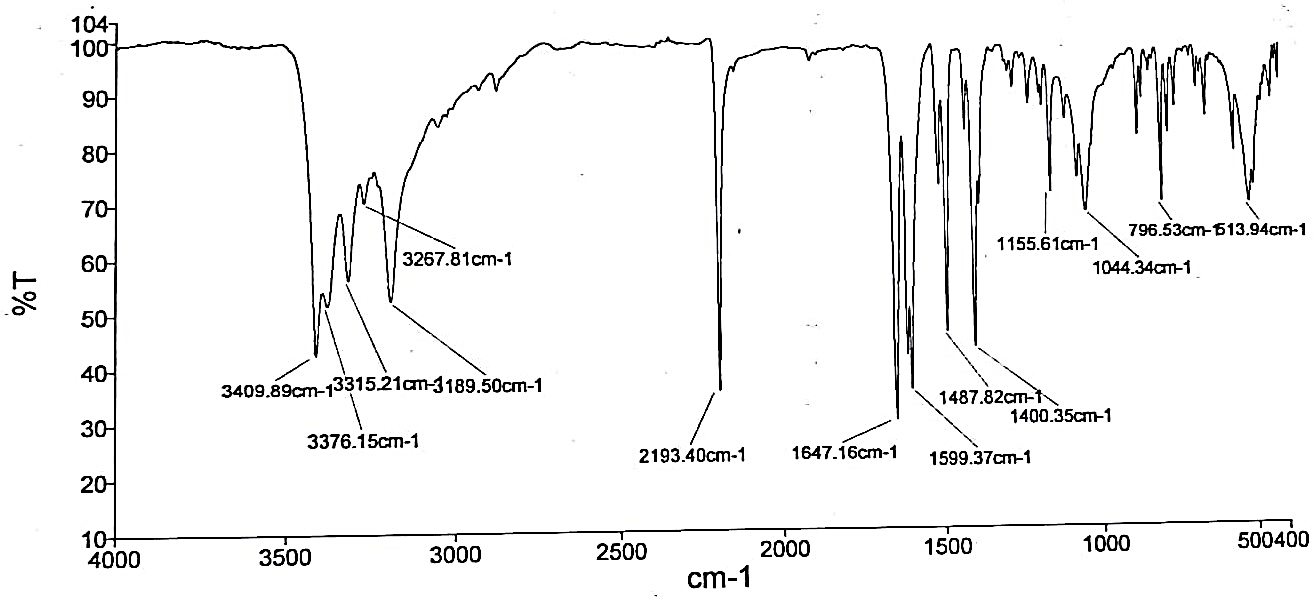
**

## IR Spectrum of *6-amino-3-methyl-4-(p-tolyl)-1,4-dihydropyrano[2,3-c]pyrazole-5-carbonitrile* (**5g**).

**
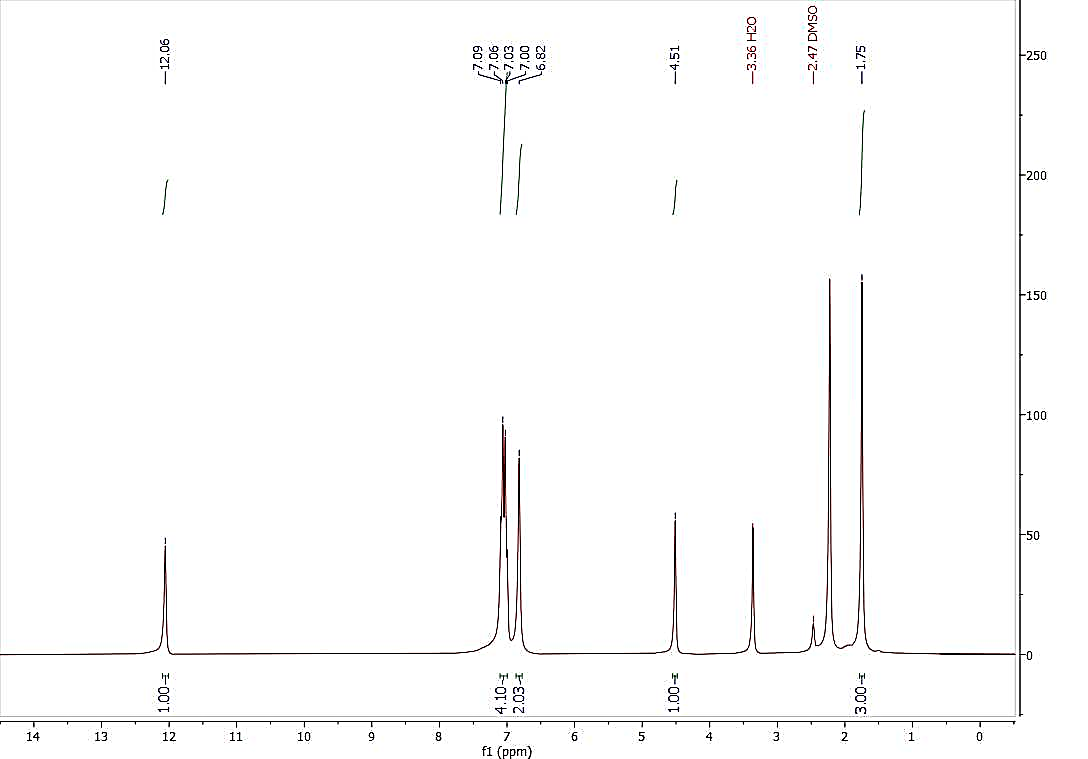
**

## HNMR Spectrum of *6-amino-3-methyl-4-(p-tolyl)-1,4-dihydropyrano[2,3-c]pyrazole-5-carbonitrile* (**5g**).

**
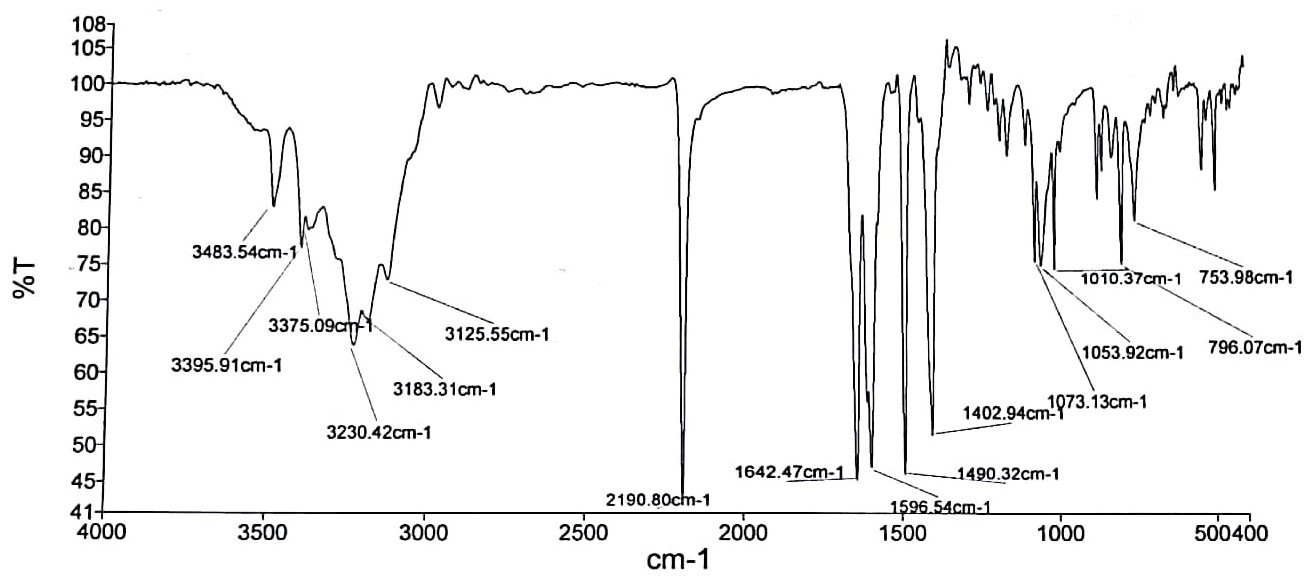
**

## IR Spectrum of *6-amino-4-(4-bromophenyl)-3-methyl-1,4-dihydropyrano[2,3-c]pyrazole-5-carbonitrile* (**5h**).

**
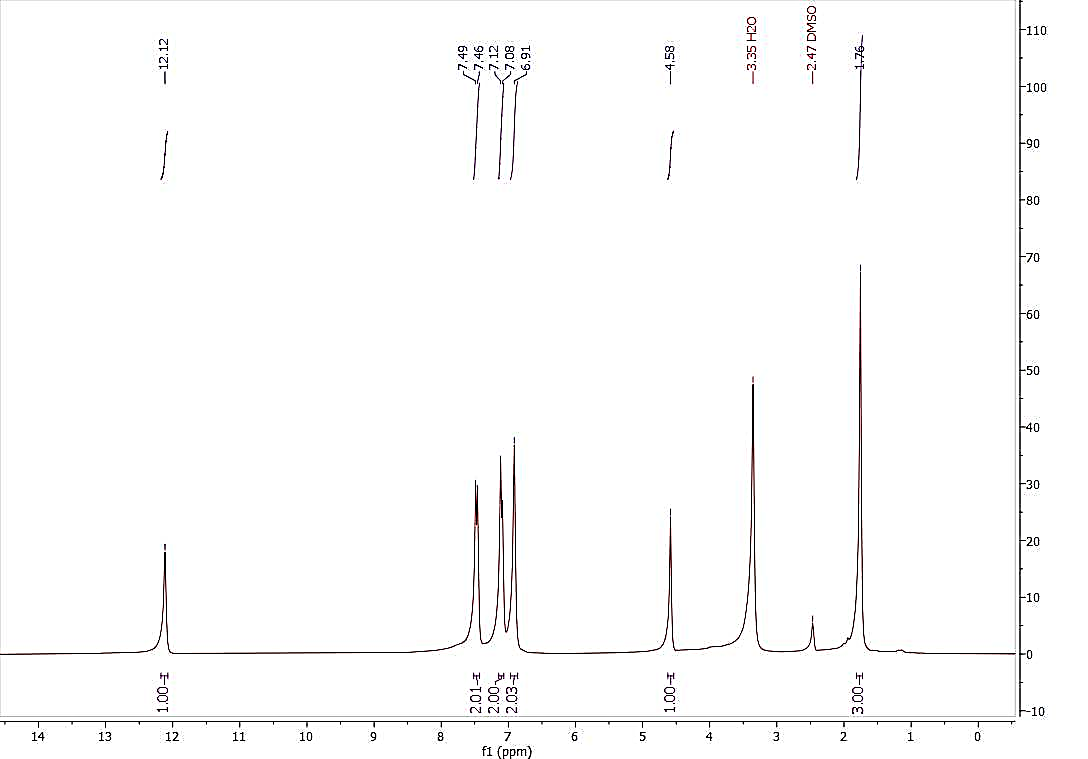
**

## HNMR Spectrum of *6-amino-4-(4-bromophenyl)-3-methyl-1,4-dihydropyrano[2,3-c]pyrazole-5-carbonitrile* (**5h**).

**
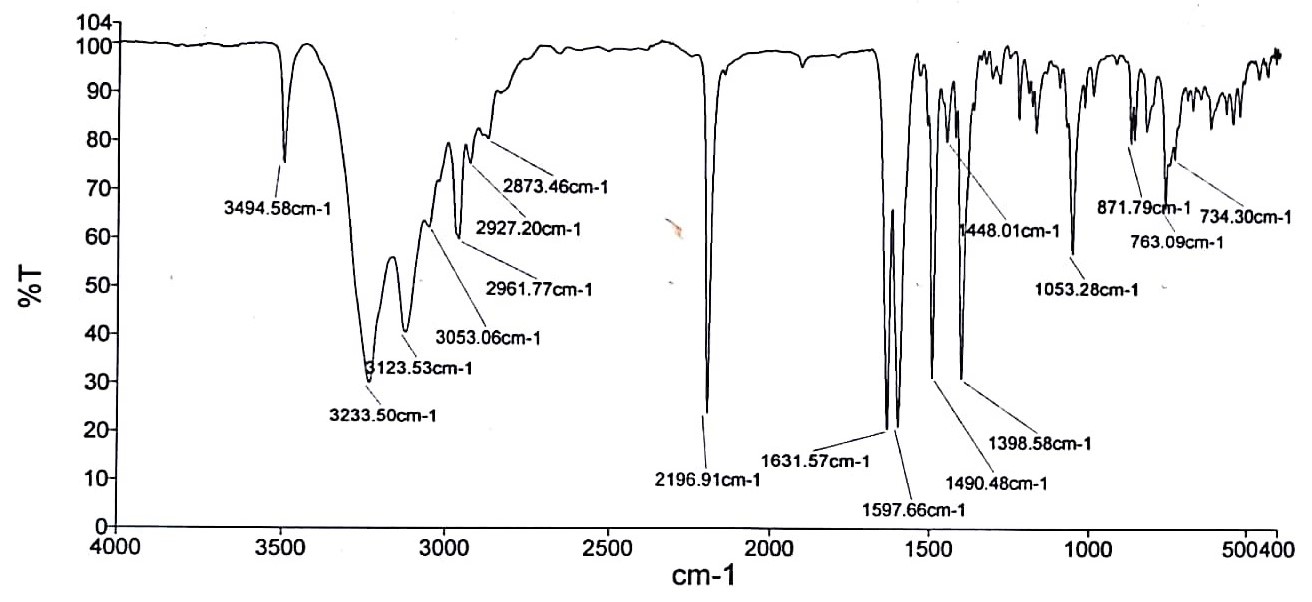
**

## IR Spectrum of *6-amino-4-(4-isopropylphenyl)-3-methyl-1,4-dihydropyrano[2,3-c]pyrazole-5-carbonitrile* (**5i**).

**
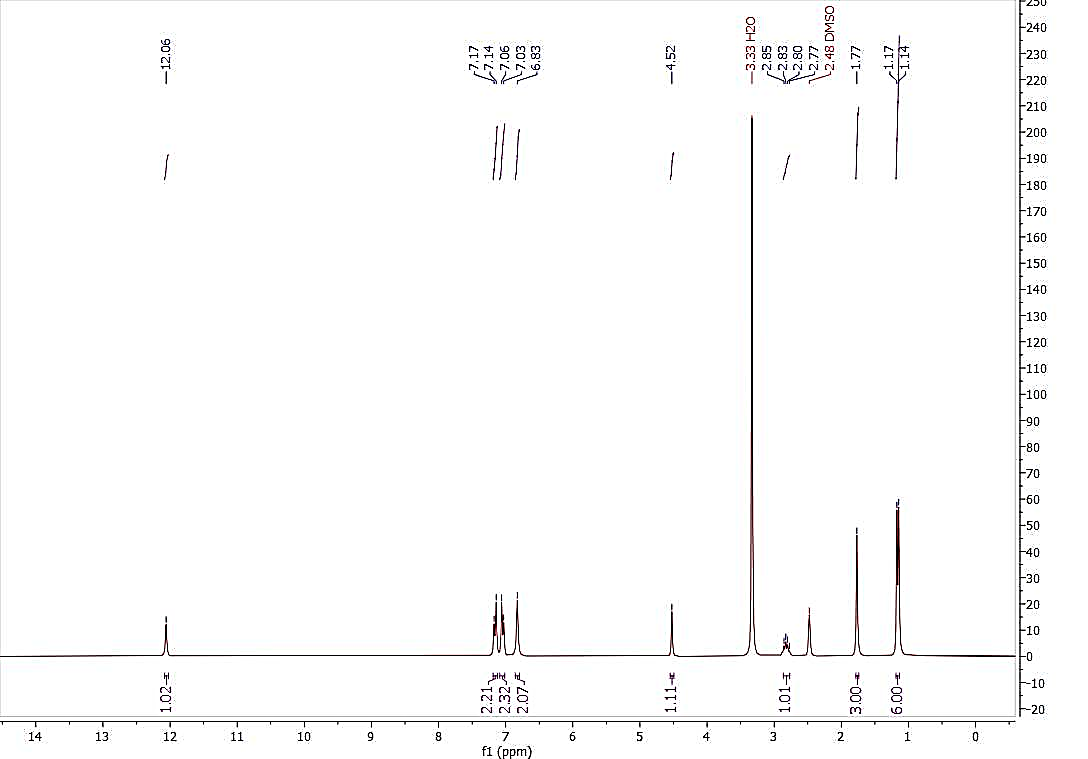
**

## HNMR Spectrum of *6-amino-4-(4-isopropylphenyl)-3-methyl-1,4-dihydropyrano[2,3-c]pyrazole-5-carbonitrile* (**5i**).

**
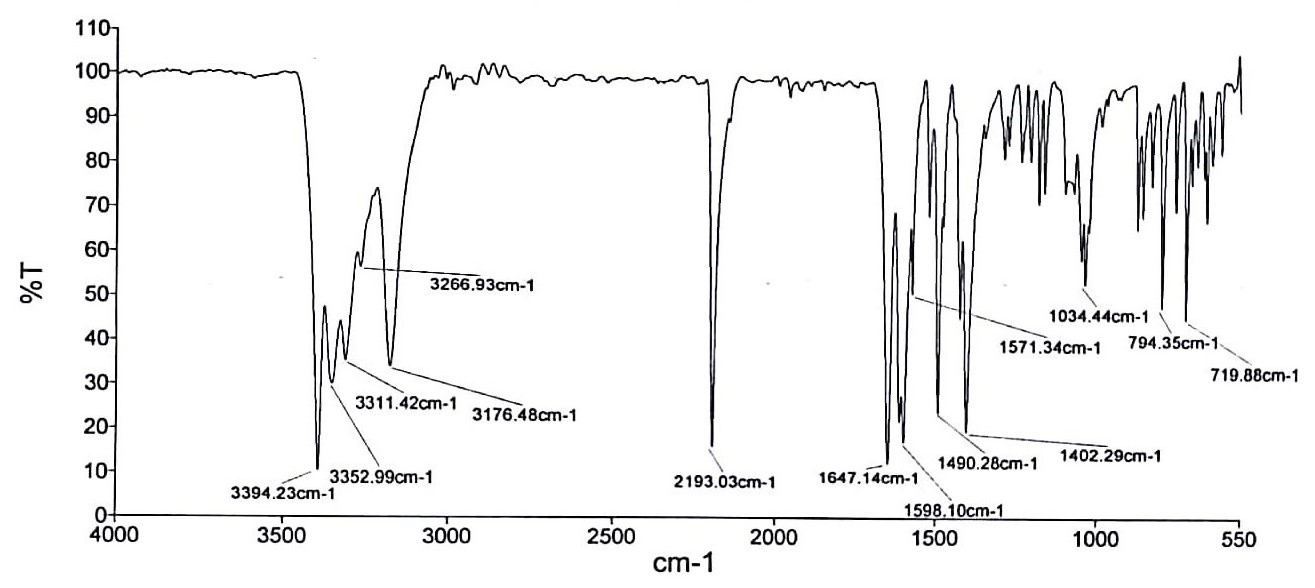
**

## IR Spectrum of *6-amino-3-methyl-4-(pyridin-3-yl)-1,4-dihydropyrano[2,3-c]pyrazole-5-carbonitrile* (**5j**).

**
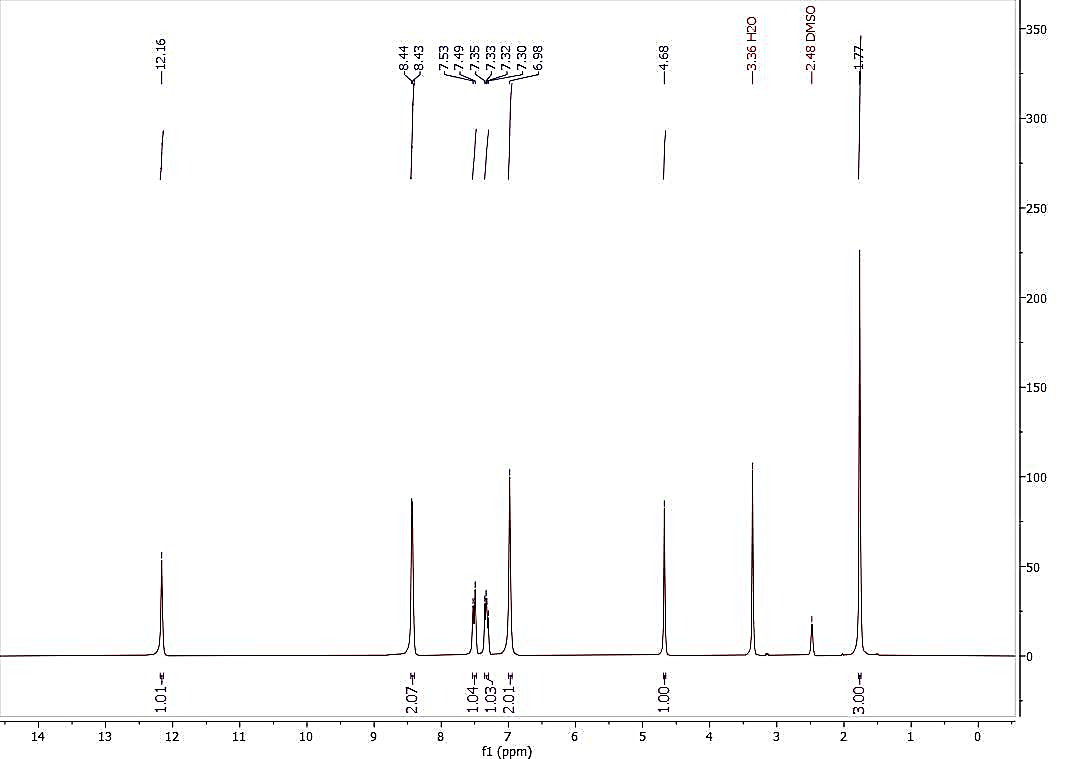
**

## HNMR Spectrum of *6-amino-3-methyl-4-(pyridin-3-yl)-1,4-dihydropyrano[2,3-c]pyrazole-5-carbonitrile* (**5j**).

**
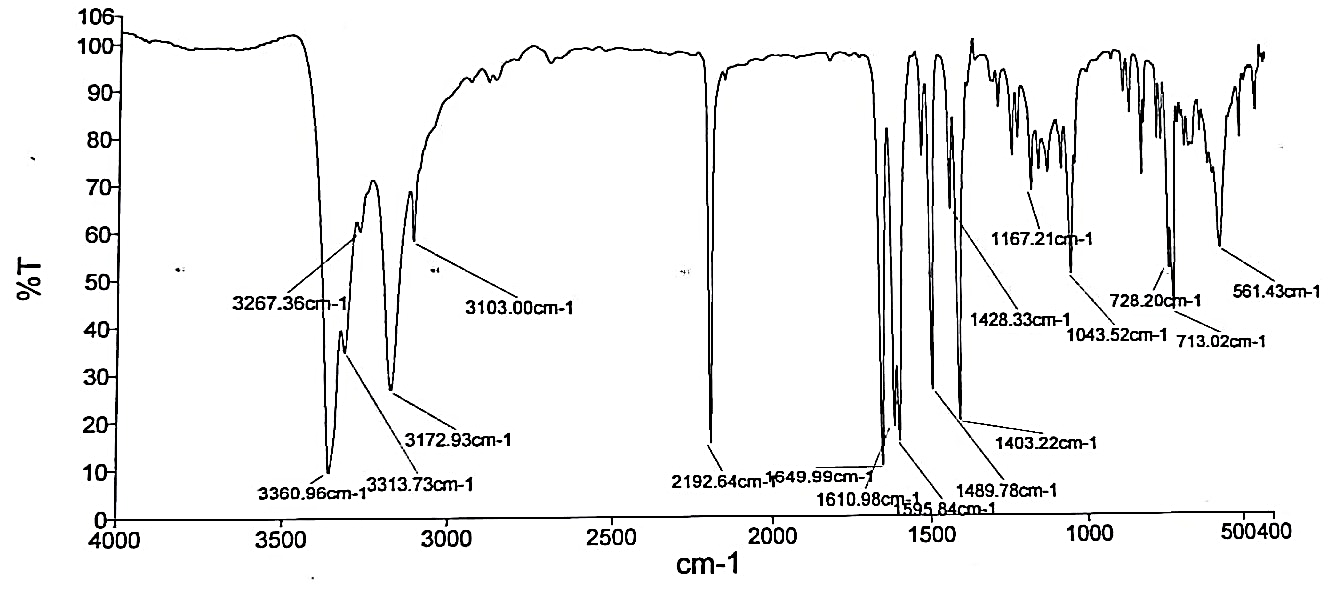
**

## IR Spectrum of *6-amino-3-methyl-4-(thiophen-2-yl)-1,4-dihydropyrano[2,3-c]pyrazole-5-carbonitrile* (**5k**).

**
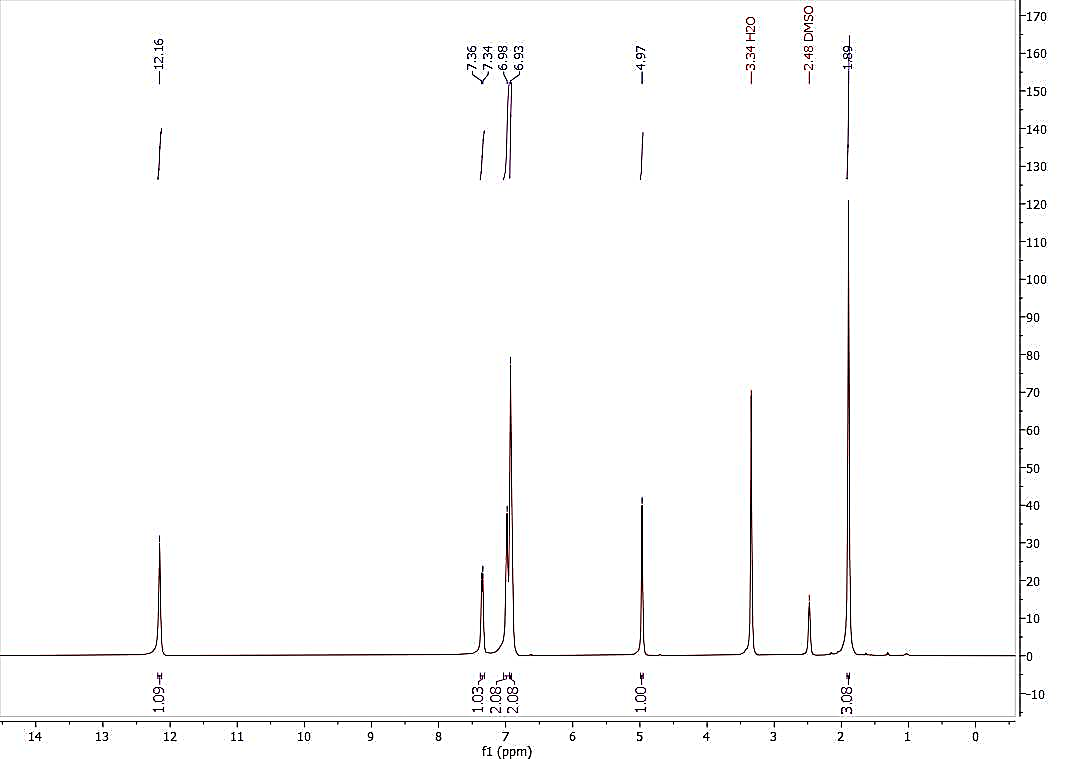
**

## HNMR Spectrum of *6-amino-3-methyl-4-(thiophen-2-yl)-1,4-dihydropyrano[2,3-c]pyrazole-5-carbonitrile* (**5k**).

**
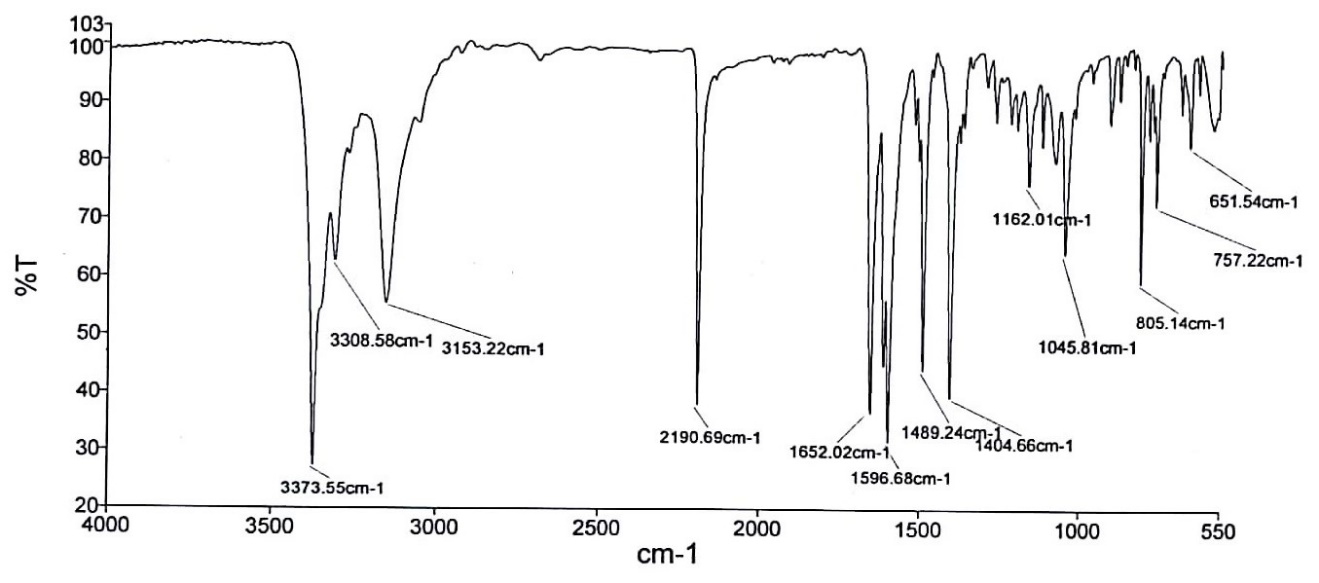
**

## IR Spectrum of *6-amino-3-methyl-4-(naphthalen-1-yl)-1,4-dihydropyrano[2,3-c]pyrazole-5-carbonitrile* (**5l**).

**
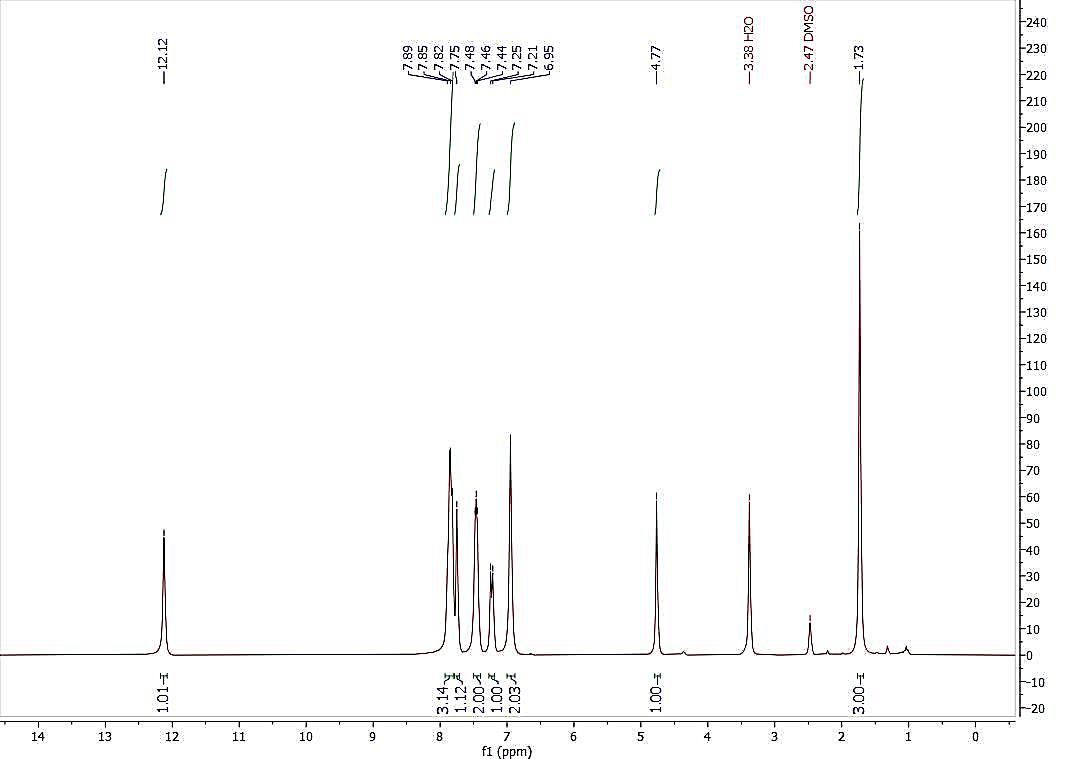
**

## HNMR Spectrum of *6-amino-3-methyl-4-(naphthalen-1-yl)-1,4-dihydropyrano[2,3-c]pyrazole-5-carbonitrile* (**5l**).

**
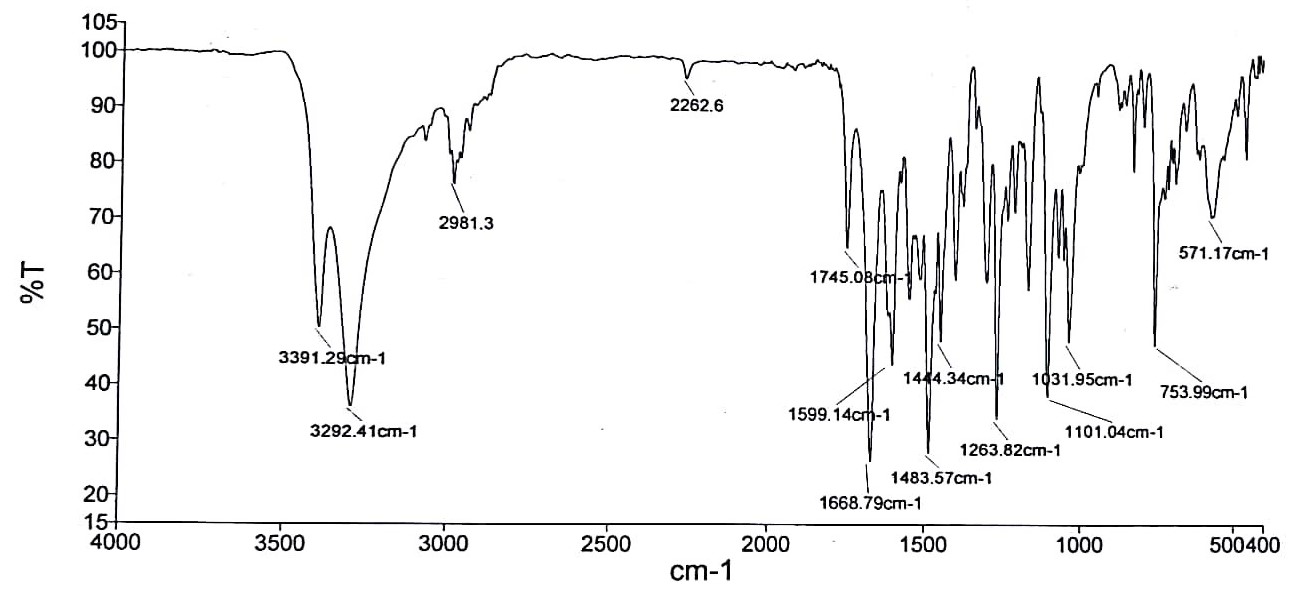
**

## IR Spectrum of *ethyl 6-amino-4-(2-chlorophenyl)-3-methyl-1,4-dihydropyrano[2,3-c]pyrazole-5-carboxylate* (**5m**).

**
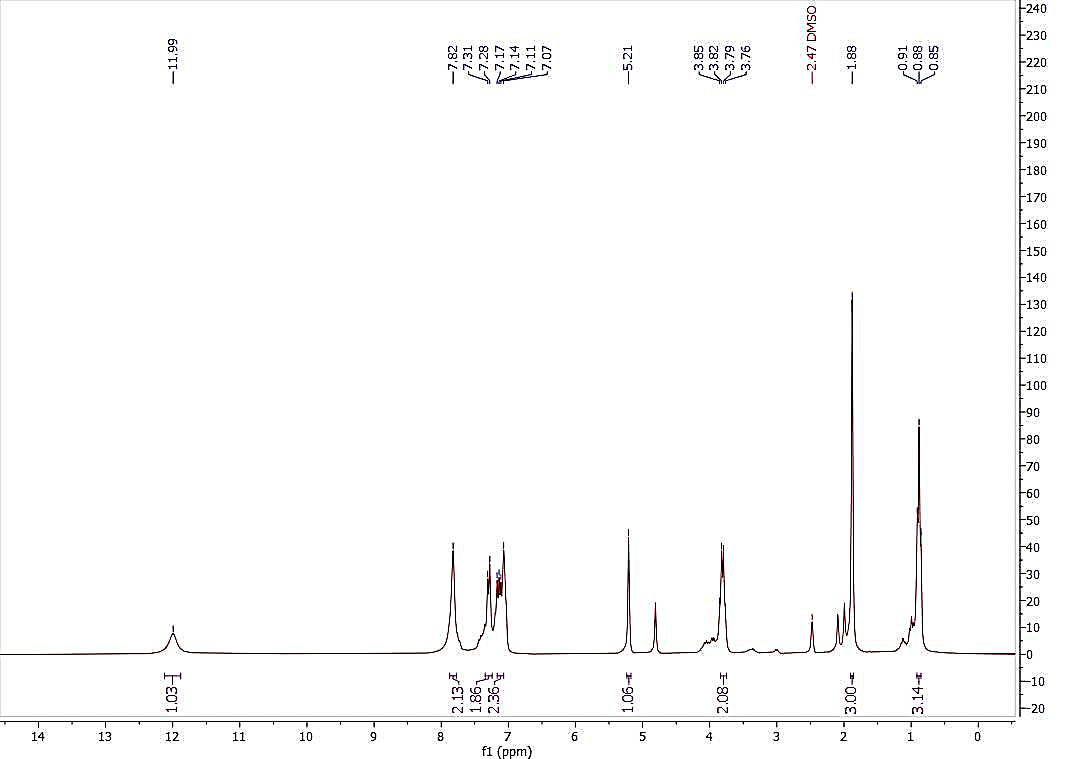
**

## HNMR Spectrum of *ethyl 6-amino-4-(2-chlorophenyl)-3-methyl-1,4-dihydropyrano[2,3-c]pyrazole-5-carboxylate* (**5m**).

**
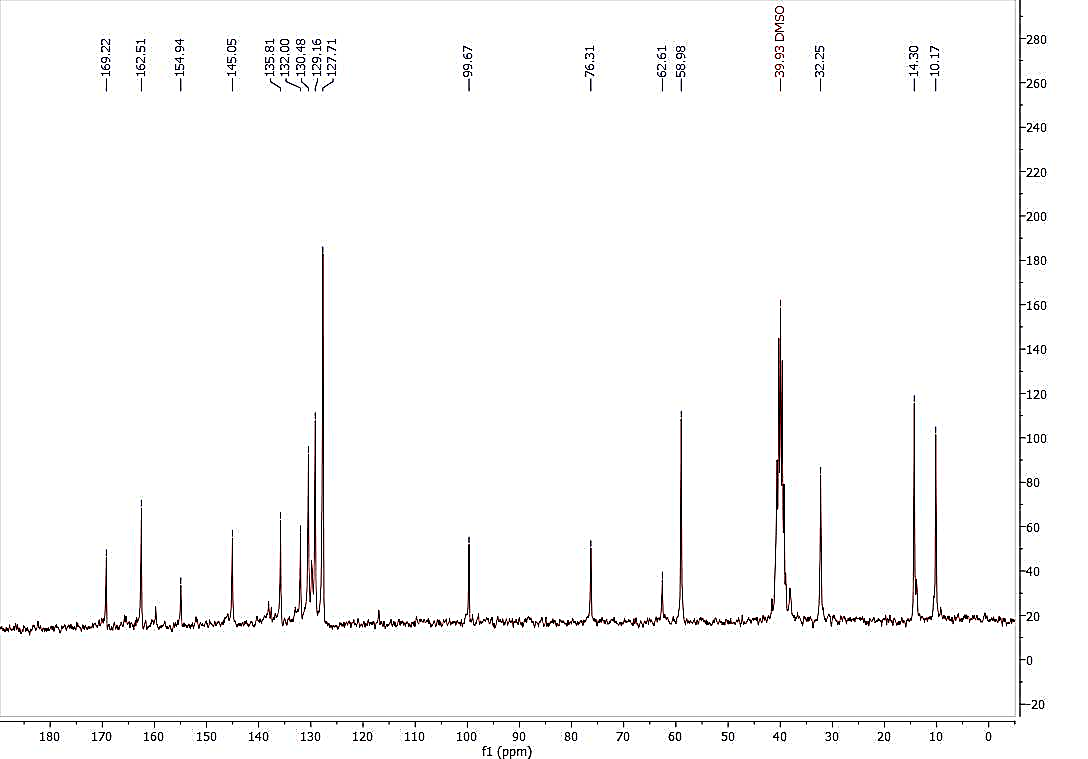
**

## CNMR Spectrum of *ethyl 6-amino-4-(2-chlorophenyl)-3-methyl-1,4-dihydropyrano[2,3-c]pyrazole-5-carboxylate* (**5m**).


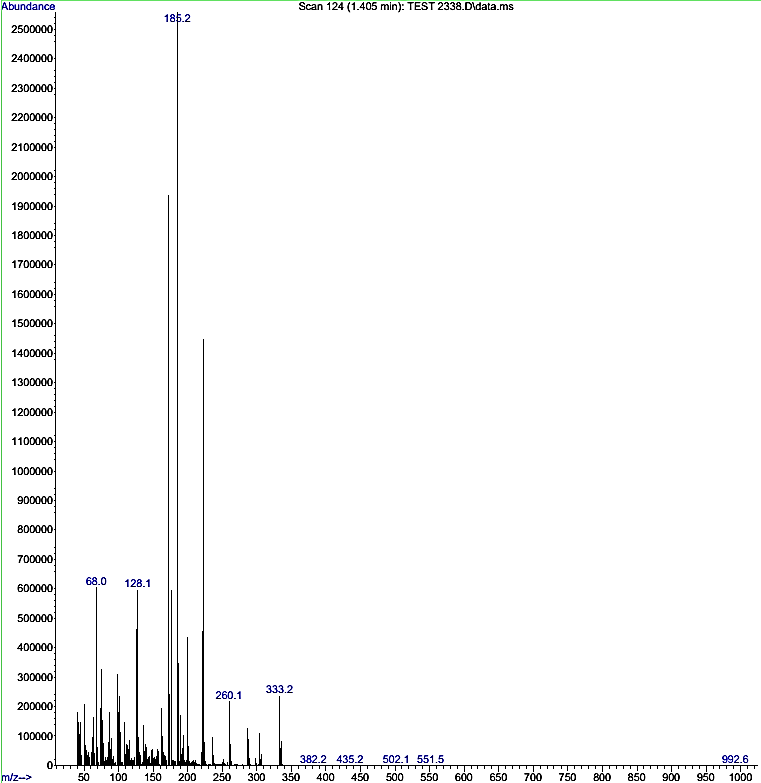


## MS Spectrum *of* *ethyl 6-amino-4-(2-chlorophenyl)-3-methyl-1,4-dihydropyrano[2,3-c]pyrazole-5-carboxylate* (**5m**).

**
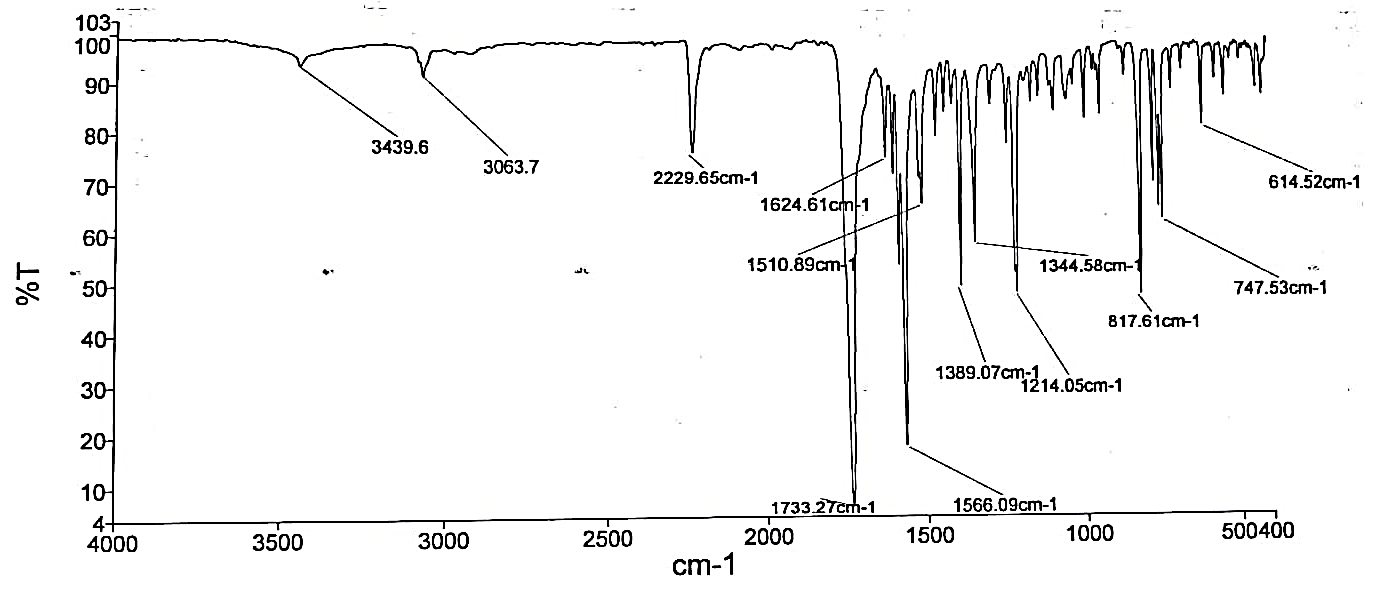
**

## IR Spectrum of *ethyl 6-amino-4-(2-hydroxynaphthalen-1-yl)-3-methyl-1,4-dihydro pyrano[2,3-c] pyrazole-5-carboxylate* (**5n**).

**
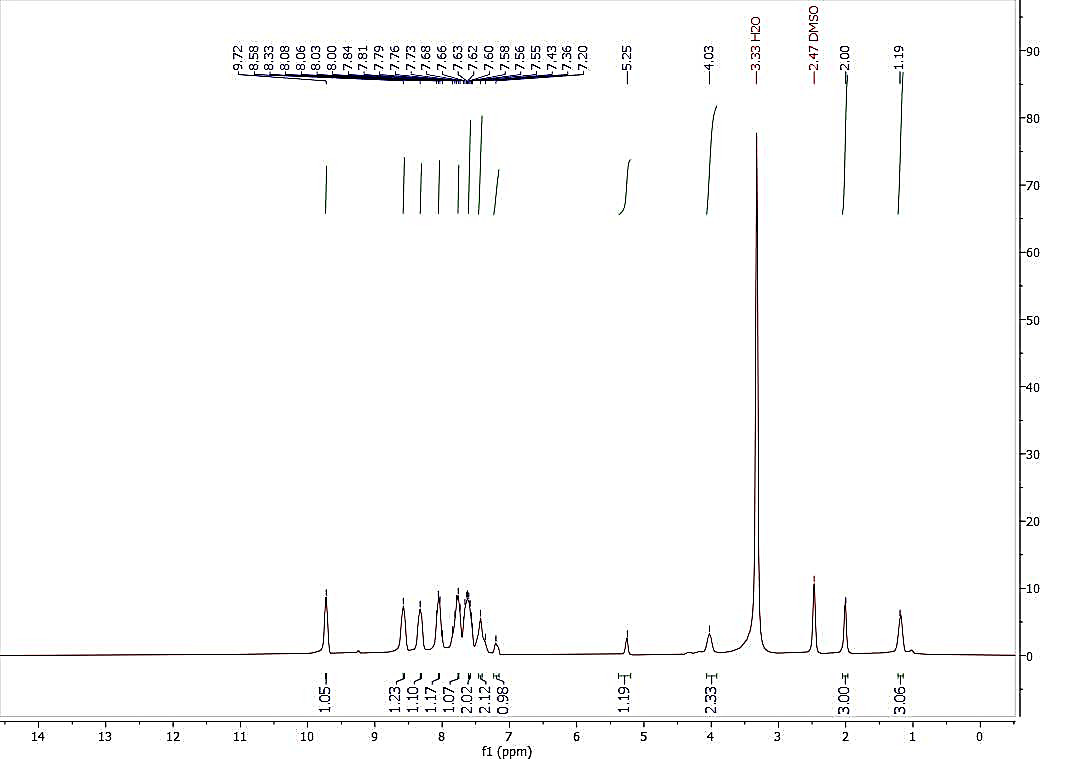
**

## HNMR Spectrum of *ethyl 6-amino-4-(2-hydroxynaphthalen-1-yl)-3-methyl-1,4-dihydro pyrano[2,3-c] pyrazole-5-carboxylate* (**5n**).

**
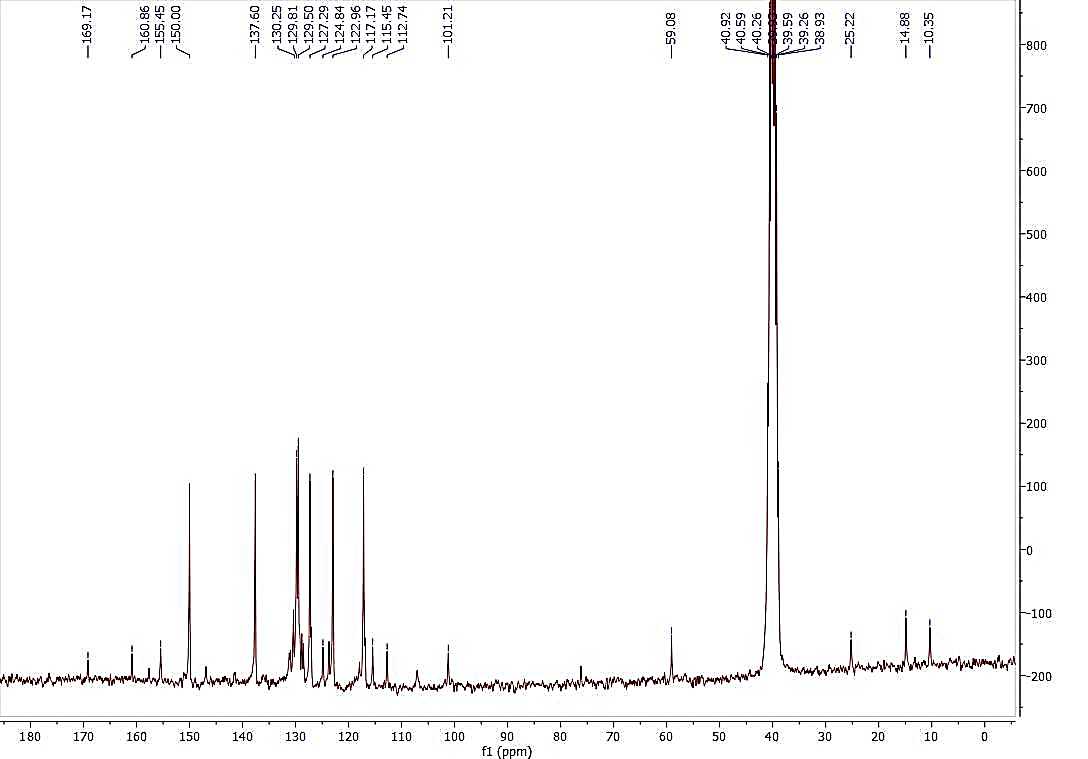
**

## CNMR Spectrum of *ethyl 6-amino-4-(2-hydroxynaphthalen-1-yl)-3-methyl-1,4-dihydro pyrano[2,3-c] pyrazole-5-carboxylate* (**5n**).


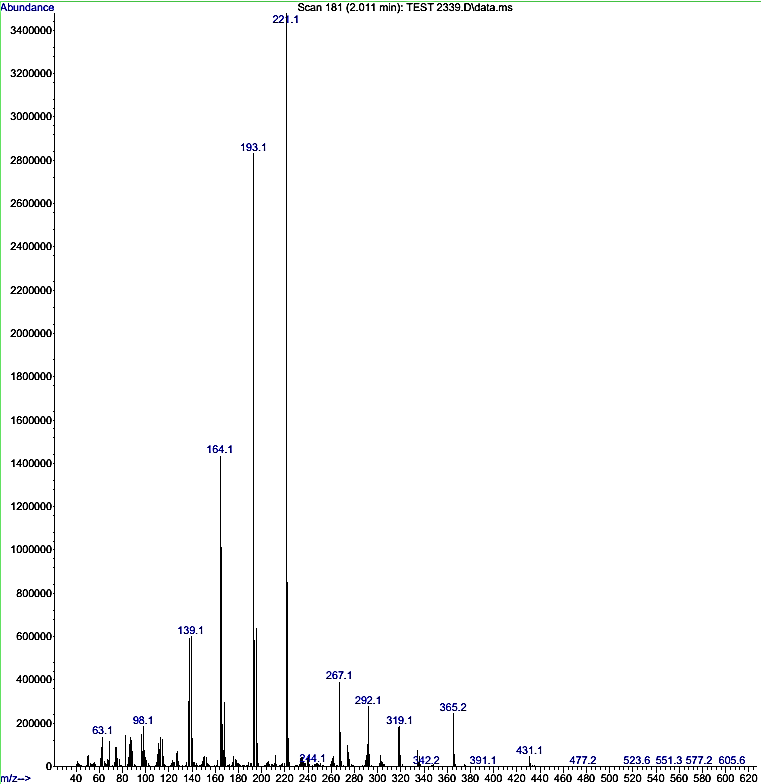


## MS Spectrum of *ethyl 6-amino-4-(2-hydroxynaphthalen-1-yl)-3-methyl-1,4-dihydro pyrano[2,3-c] pyrazole-5-carboxylate* (**5n**).

**
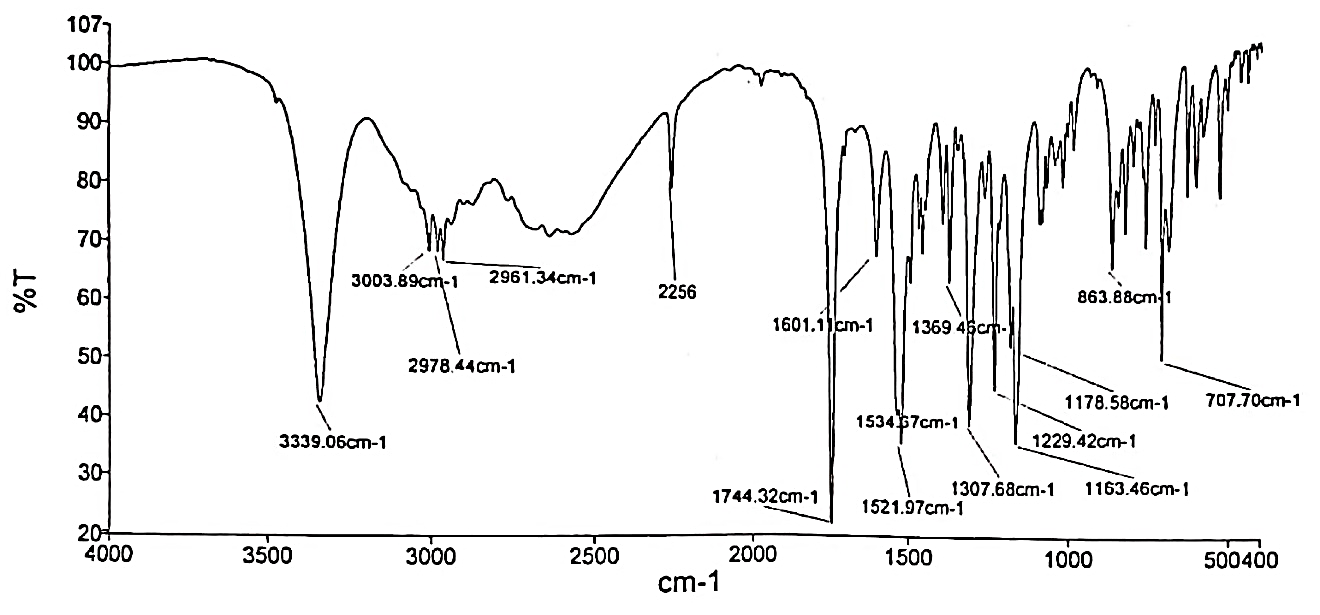
**

## IR Spectrum of *ethyl 4-benzyl-5-imino-3-methyl-4,5-dihydro-1H-furo[2,3-c]pyrazole-4-carboxylate* (**5o**).


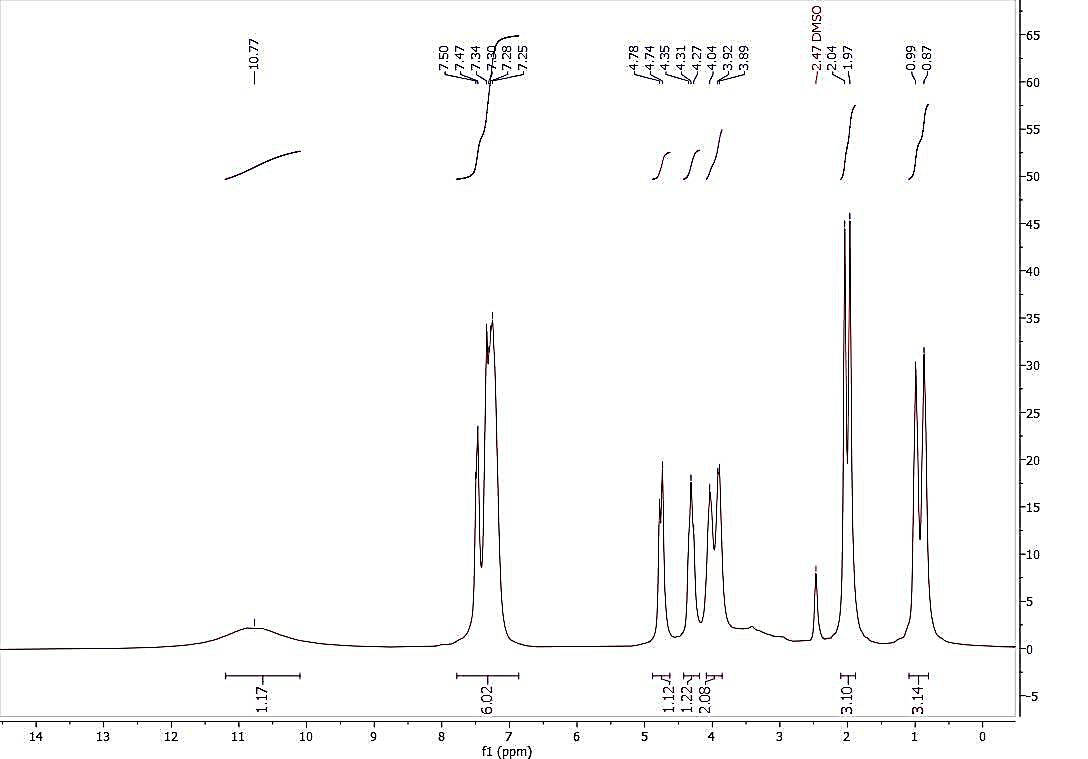


## HNMR Spectrum of *ethyl 4-benzyl-5-imino-3-methyl-4,5-dihydro-1H-furo[2,3-c]pyrazole-4-carboxylate* (**5o**).


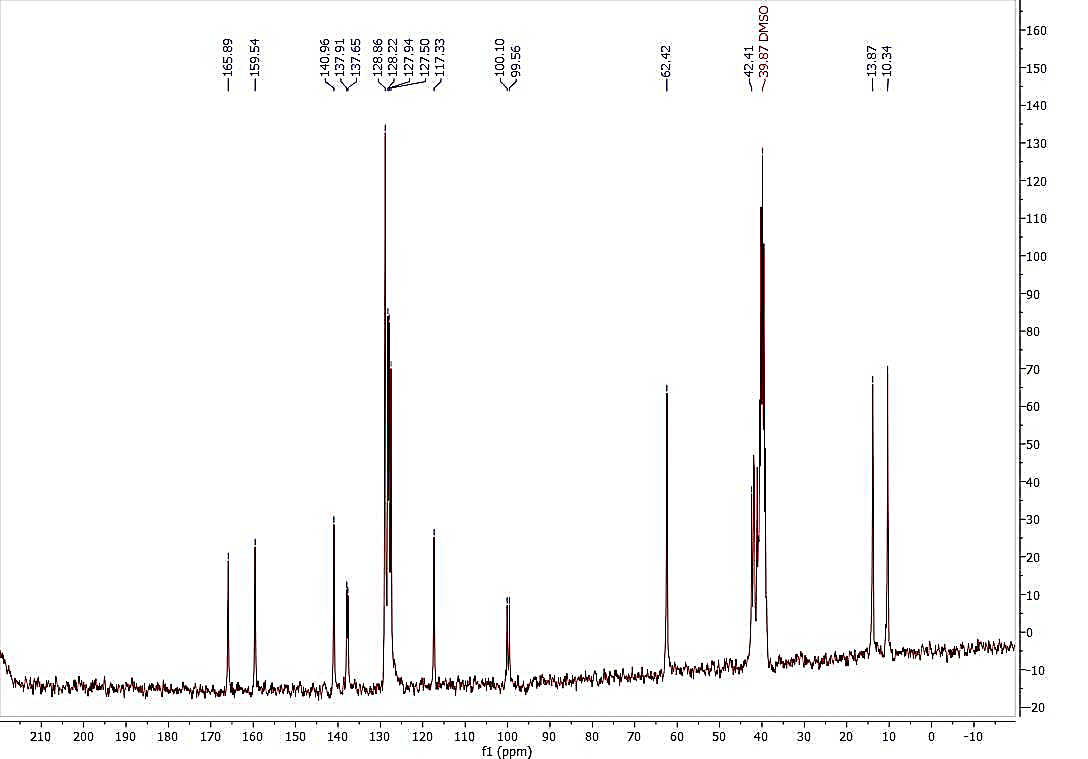


## CNMR Spectrum of *ethyl 4-benzyl-5-imino-3-methyl-4,5-dihydro-1H-furo[2,3-c]pyrazole-4-carboxylate* (**5o**).


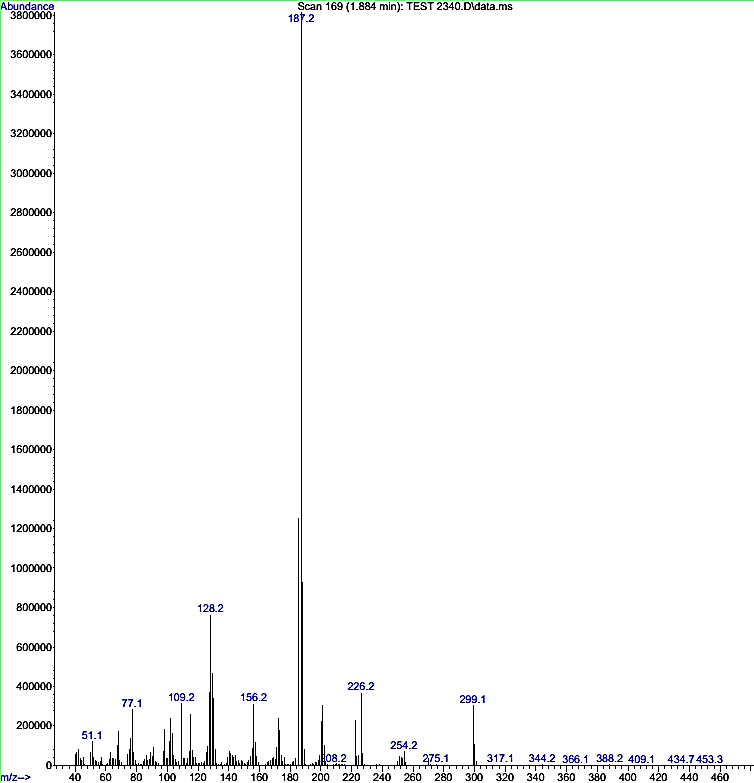


## MS Spectrum of *ethyl 4-benzyl-5-imino-3-methyl-4,5-dihydro-1H-furo[2,3-c]pyrazole-4-carboxylate* (**5o**).
